# Incorporating crystalline smart materials to fabricate 4D printed photomechanical actuators with photovoltaic performance

**DOI:** 10.1002/smo2.70026

**Published:** 2025-11-24

**Authors:** Yujie Liu, Jinjin Liu, Liqin Hao, En Lin, Jiaxi Wang, Tonghai Wang, Shubo Geng, Peng Cheng, Zhenjie Zhang

**Affiliations:** ^1^ College of Chemistry State Key Laboratory of Medicinal Chemical Biology Nankai University Tianjin China; ^2^ Frontiers Science Centre for New Organic Matter Key Laboratory of Advanced Energy Material Chemistry (Ministry of Education) Nankai University Tianjin China; ^3^ Nankai International Advanced Research Institute (Shenzhen Futian) Nankai University Tianjin China

**Keywords:** 4D printing, COFs, photomechanical actuators, photovoltaic power generation, smart materials

## Abstract

Fabricating macroscale smart actuators that can convert light energy into other forms of energy, especially mechanical and electrical energy, is of great significance. Herein, a simple and efficient 4D printed method for fabricating photomechanical actuators based on micro/nano‐scale crystals is developed. The high versatility and generality of this method are successfully demonstrated using nine different types of photoresponsive crystalline actuators, including acylhydrazone‐, anthracene‐, olefin‐, and azobenzene‐based molecular crystals and covalent organic frameworks (COFs). The low‐cost neutral silicone sealant elastomer is first chosen as the photomechanical 4D printing matrix. Notably, these actuators can be used to perform bionic motions (the first windmills spin using crystalline material, dragonflies fly, and sunflowers bloom) under the stimulation of visible light and can realize energy conversion from mechanical energy into electricity when coupled with a piezoelectric membrane. This work provides new insights into the design and manufacturing of smart photomechanical actuators and electricity generators and expands the application scope of COFs.

## Graphical abstract

A versatile 4D printed approach was developed to fabricate complex and refined photomechanical actuators using crystalline materials, including molecular crystals and covalent organic frameworks. The prepared smart actuators exhibited outstanding photomechanical and photovoltaic performance.

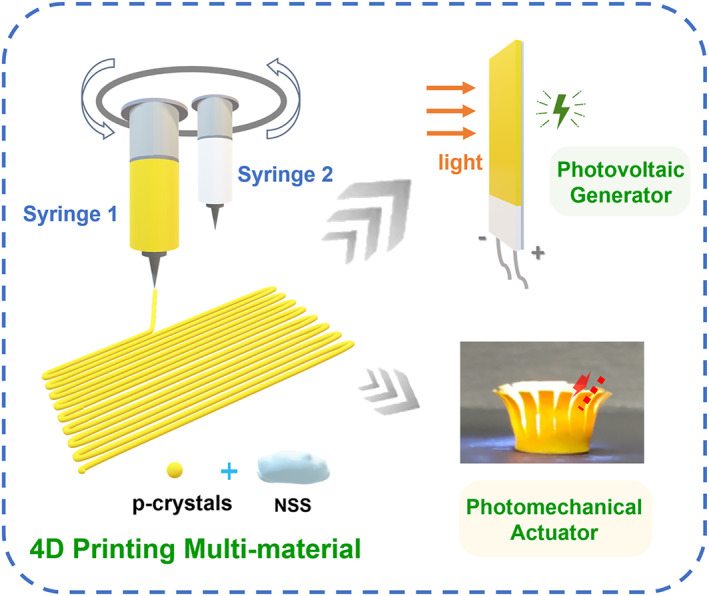

## 1 INTRODUCTION

Mechanically responsive smart materials, which can convert chemical energy into mechanical movement, have captured researchers' attention in various advanced applications such as biomimetics, soft robotics, and mechanical actuators.[^1^, ^2^, ^3^, ^4^, ^5^, ^6^, ^7^, ^8^, ^9^, ^10^, ^11^, ^12^, ^13^] Upon environmental stimuli such as light,[^1^, ^14^] temperature,[^15^, ^16^] electricity,^[17]^ and pH,[^18^, ^19^] reversible deformation of mechanical responses for smart materials can be realized, such as bending, twisting, jumping, rolling, contraction, expansion, and fragmentation.[^2^, ^3^, ^16^, ^20^] Among these stimuli, light has numerous benefits, including cost‐effectiveness, wireless‐driven, additionally noninvasive, rapid response, and a multitude of wireless remote techniques (e.g., adjusting light wavelength, intensity, polarization, and irradiation position). As a result, photomechanical materials have demonstrated great application potential.[^14^, ^21^, ^22^, ^23^, ^24^, ^25^] The current work mostly concentrates on photomechanical materials with low or no crystallinity, such as organic polymers, hydrogels, and liquid crystals.[^26^, ^27^] By contrast, crystalline materials with well‐defined and regular molecular arrays (e.g., molecular crystals, framework materials) have not been intensively studied until recently, but they offer a new opportunity to create novel smart materials with improved performance.^[28]^ Crystalline materials with precisely determined structures and ordered molecular packing can provide rapid response, faster relaxation recovery, a higher Young's modulus, and a clearer photomechanical mechanism.[^29^, ^30^, ^31^, ^32^, ^33^] Nevertheless, owing to their small size, fragility, and poor mechanical characteristics, crystalline materials are not able to convert large enough energy into practical work or fabricating macroscale devices. Given these circumstances, combining crystalline materials with connective polymers to create hybrid systems is an effective strategy for resolving the above challenges but remains underexplored.[^29^, ^34^, ^35^] Recently, the emerging 4D printing technology that adds the dimension of time to 3D printing has attracted increasing attention because it can print objects that can change shape or function over time in response to various external stimuli. Thus, 4D printing can combine 3D printing with “smart materials” that have great potential in various application fields, including medicine, aerospace, architecture, etc.[^36^, ^37^, ^38^, ^39^, ^40^, ^41^, ^42^, ^43^, ^44^, ^45^, ^46^, ^47^, ^48^]

In this work, we developed a new 4D printing technology by mixing neutral silicone sealant (NSS) with photoresponsive crystals to fabricate photomechanical actuators. NSS is an inexpensive and accessible polymer perfectly adapted to the requirements of photomechanical 4D printing materials for high transparency (allowing the smooth entry of light to trigger photoresponse), high elasticity (helping the actuator to recover quickly after the response), high strength (increasing the mechanical strength of the actuator to facilitate large deformation and recyclability), and easy molding (self‐supporting features avoid the use of additional supporting materials). NSS for 4D printing also does not need organic solvents to mix with the crystalline materials, protecting the crystals against dissolution to achieve their photoresponsive performance perfectly.[^49^, ^50^, ^51^, ^52^, ^53^] Here, we investigated a series of photomechanical crystalline materials, including various types of molecular crystals and covalent organic frameworks (COFs). Incorporating these crystalline materials with NSS afforded a series of 4D photomechanical actuators based on direct ink writing. The printed photomechanical actuators such as windmills, dragonflies, and sunflowers can imitate the biological characteristics of nature for light responses such as rotating, opening, and closing under the stimulation of an uneven unilateral light source. Then, we fabricated bilayer devices by coupling photoresponsive actuators with piezoelectric films to output stable power upon periodic light stimuli.

## 2 RESULTS

### 2.1 Design and preparation of p‐crystal @ NSS actuators

**FIGURE 1:**
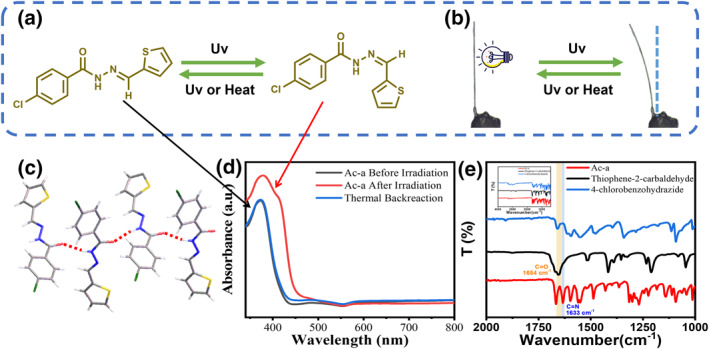
Structure and photomechanical properties. (a) Illustration of the reversible structure *E‐Z* isomerization around the ‐C=N‐ bond of Ac‐a triggered by UV light irradiation. (b) The reversible bending behavior of Ac‐a crystal. (c) Hydrogen bonding synthon present in the crystal structures of Ac‐a. (d) UV‐Vis spectra of Ac‐a before and after UV light irradiation and thermal reaction. (e) FT‐IR spectra of Ac‐a and monomers.

To demonstrate the proof of concept, we designed and synthesized a new type of acylhydrazone derivative crystals as the research object, which can undergo reversible *E‐Z* isomerization around the ‐C=N‐ bond, leading to a change in its configuration under light irradiation and heating.^[54]^ The Ac‐a crystal was then prepared as a representative (Figure 1a, Supporting Information S1: Supporting Information S1: Figure S1), FT‐IR spectra showed the disappearance of the characteristic absorption peaks C=O at 1654 cm^−1^ of aldehyde monomers, and C=N bond peaks appeared at 1633 cm^−1^ (Figure 1e). After crystallization in acetonitrile, Ac‐a formed needle‐like crystals (Supporting Information S1: Figure S8). Powder X‐ray Diffraction measurements revealed that the bulky samples possessed characteristic patterns consistent with the calculated patterns, indicative of their high purity (Supporting Information S1: Figure S9). Single crystal X‐ray diffraction analysis revealed that Ac‐a crystallized in the monoclinic space group *P*2_1_ */c*. The molecules are linked by N‐H…O hydrogen‐bonds (N…O distance of 2.87 Å) to form one dimensional chains in the crystal structure (Figure 1c, Supporting Information S1: Table S1). Then, we studied the photoresponsive characteristics of the Ac‐a crystals. As shown in Figure 1b, Ac‐a crystals fixed onto glass fiber could gradually bend away from UV light sources in 3 s. When irradiating from the opposite direction or by heating, crystals can bend back to the initial position. The mechanism of these photomechanical motions was then investigated. The changes in the absorption peak were observed through UV‐vis spectra, which were related to the reversible *E‐Z* isomerization around the ‐C=N‐ bond (Figure 1a,d).

**SCHEME 1:**
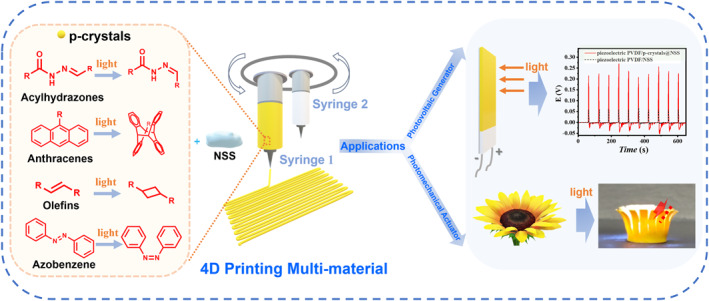
Design and preparations. Illustration of the process of preparing 4D printed photomechanical actuators as well as photovoltaic generators.

**FIGURE 2:**
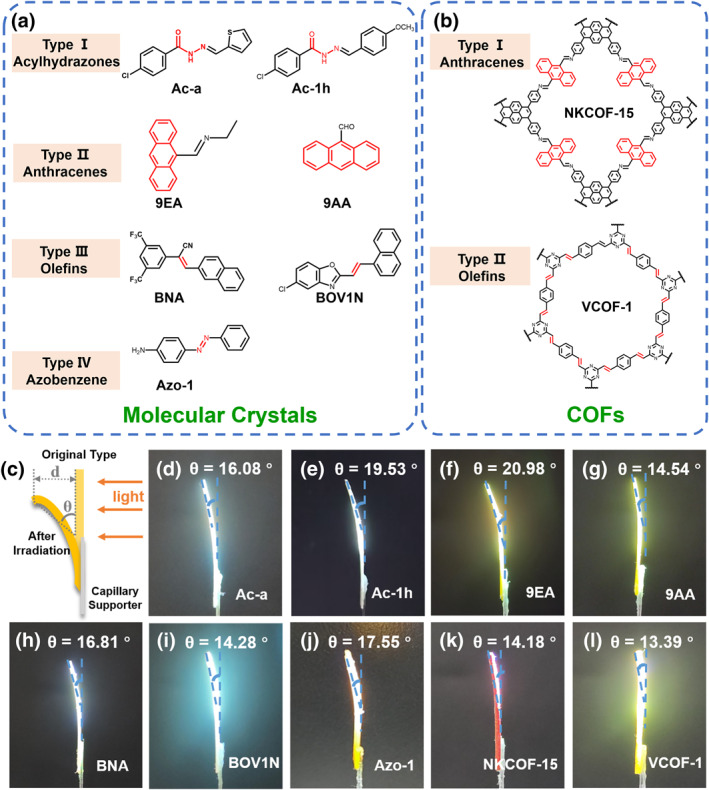
Generality characterization. (a) Molecular structures of Ac‐a, Ac‐1h, 9EA, 9AA, BNA, BOV1N, and Azo‐1. (b) Molecular structures of NKCOF‐15, VCOF‐1. (c) Schematic illustration of the defined displacement (*d*) and bending angle (*θ*) in the mechanically responsive process. (d‐l) Illustration of the photoresponsive bending behavior of p‐crystals@NSS actuators. NSS, neutral silicone sealant.

We fabricate macroscale hybrid photomechanical actuators using 4D printing technology with NSS as the matrix of the printing ink. The process of preparing p‐crystals@NSS inks (p‐crystals representing photomechanical crystals), controllable 4D printed photomechanical actuators, and photovoltaic generators is schematically shown in Scheme 1. Firstly, the Ac‐a crystals were mixed with the NSS to obtain printing inks for Ac‐a@NSS. A high‐speed ball milling method was performed to achieve uniformly doped printing ink (Supporting Information S1: Figure S10). Then, the Ac‐a@NSS inks were loaded inside the plastic syringe. Adjusting the *Z*‐axis (syringe holder), *X*‐ and *Y*‐axes (stage) were used to place the syringe needle proximal to the substrate on the stage. The pressure for ideal printing was optimized by checking the continuity and stability of the hydrogel extruded from the needle. Finally, Ac‐a@NSS was extruded, and the ink was shear‐thinning, which quickly recovered to high viscosity for shape retention. Notably, p‐crystals@NSS inherited the photomechanical behavior of the p‐crystals, which indicated that these actuators could bend away from light and recover their original shape upon keeping it at room temperature. It was found that the large amount of NSS doped into p‐crystals did not affect the photomechanical performance of the actuator, which showed a faster response (Figure 2d). We used the displacement (*d*) and bending angle (*θ*) to evaluate the degree of the mechanical response process (Figure 2c). The bending angle *θ* of Ac‐a@NSS actuators at the illumination distance (*d* = 2 cm) and illumination time (*t* = 3 s) was 16.08° when the molar ratio of NSS and Ac‐a was 50:1 (Ac‐a@NSS‐50) (Figure 2d). For comparison, the pure SiO_2_@NSS‐50 was also printed as the blank, which did not exhibit any light response (Supporting Information S1: Figure S11). These results demonstrated that the photomechanical behavior of the hybrid materials originated from the doped Ac‐a.

**FIGURE 3:**
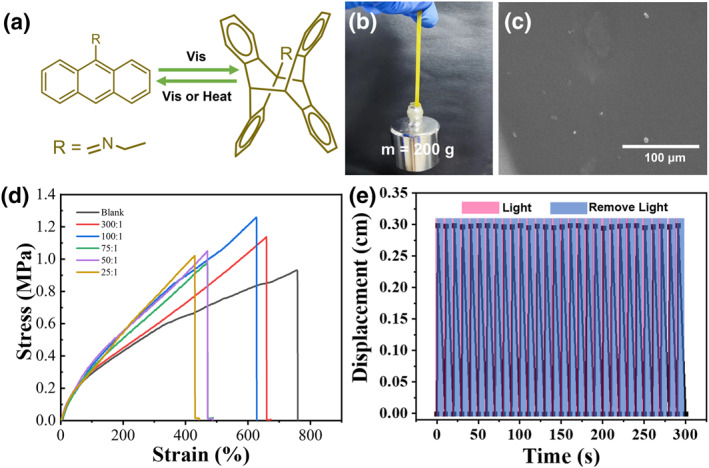
Physical characterization of 9EA@NSS‐50 actuator. (a) Illustration of the reversible structure transformation ([4 + 4] photodimerization) of 9EA triggered by visible light irradiation. (b) Images of the 9EA@NSS‐50 after being stretched by 200 g weights. (c) The SEM image of 9EA@NSS‐50. (d) Stress−strain curves of 9EA@NSS actuator with different content of 9EA in the printing ink. (e) Plot of the repeating cycles of 9EA@NSS‐50 with light and removing light. NSS, neutral silicone sealant; SEM, scanning electron microscopy.

To prove the generality of this new approach to fabricate hybrid photomechanical actuators, we explored other types of photoresponsive crystalline materials. Anthracene derivative crystals can undergo reversible [4 + 4] dimerization under light irradiation and heating accompanied by volume expansion or contraction, olefin derivatives crystals can undergo [2 + 2] photocycloaddition reactions after light irradiation, and azobenzene derivatives crystals can undergo *E‐Z* isomerization after light irradiation. One acylhydrazone derivative molecular crystals (Ac‐1h^[55]^), two anthracene derivative molecular crystals (9EA,^[34]^ 9AA^[56]^), two olefin derivative molecular crystals (BNA,^[57]^ BOV1N^[58]^), one azo derivative molecular crystal (Azo‐1^[59]^), one anthracene derivative microcrystalline COF (NKCOF‐15^[60]^), one derivative of olefin‐linked microcrystalline COF (VCOF‐1^[61]^), a total of nine types of photoresponsive crystalline materials in six categories was obtained according to literature (Figure 2a,b and Supporting Information S1: Figure S2–S7). After crystallization in the corresponding solvents, Ac‐1h, 9EA, 9AA, BNA, and BOV1N formed needle‐like crystals, Azo‐1 formed sheet‐like crystals, and NKCOF‐15 and VCOF‐1 formed microcrystalline powders (Supporting Information S1: Figures S12 and S13). Powder X‐ray Diffraction measurements revealed that Ac‐1h, 9EA, 9AA, BNA, BOV1N, Azo‐1, NKCOF‐15, and VCOF‐1 possessed the reported crystal structures and high purity (Supporting Information S1: Figure S14). The photoresponsive performance was then studied by irradiating with the UV or visble light sources in 3 s (Supporting Information S1: Figure S15). The changes in the photocycloaddition and cis‐trans isomerism of the crystals before and after light irradiation were confirmed by UV–vis, FT‐IR, Single crystal X‐ray diffraction and NMR spectroscopy (Figure 3a, Supporting Information S1: Figures S16–S20). Following the aforementioned experimental procedures, we successfully printed different p‐crystals@NSS‐50 hybrid materials, which possess good mechanical properties and show excellent photoresponsive performance as well. As shown in Figure 2e–l, the bending angle of the p‐crystals@NSS actuator driven by light ranges from 13.39° to 20.98° under the same illumination distance (*d* = 2 cm) and illumination time (*t* = 3 s) as well as the same doping ratio (p‐crystals‐50). These results further proved the universality and versatility of the p‐crystals@NSS hybrid actuator approach. It is worth mentioning that the hybrid materials obtained from the two types of COF microcrystals with photomechanical response also possess good photoresponsive properties, which prove the generality of this new method to fabricate hybrid photomechanical actuators and expand the potential application scopes of COF materials. Since 9EA@NSS‐50 exhibits the best photoresponsive performance, it was chosen as the representative example for printing condition optimization, performance characterization, and further photomechanical actuator and photovoltaic performance.

Considering the content of p‐crystals in ink significantly affects its printing parameters and final driving performance, we systematically studied the variations in the photomechanical behavior of actuators printed in ink containing varying molar ratios of p‐crystals. The bending angle *θ* of 9EA@NSS actuators gradually increases and then decreases as the molar content of the photoresponsive crystal 9EA in the ink increases at the same illumination distance (*d* = 2 cm) and illumination time (*t* = 3 s). It gradually increases from 4.6° to 20.98° and then decreases to 20.25°, with the corresponding amplitude being most prominent when the doping ratio is 9EA@NSS‐50 (Supporting Information S1: Figure S21). The reversible bending process of 9EA@NSS‐50 could be repeatedly cycled by bending away from visible light sources for 1 s to respond and keeping it at room temperature for about 9 s to recover, which could repeat 30 times within 5 min (Figures 2f and 3e, Supporting Information S1: Figures S22 and Video S1).

We also studied the influence of different printing pressures, extrusion rates, needle diameters, and number of printing layers on the printed material's morphology (Supporting Information S1: Figure S23). The optimal printing parameters for this system were determined by analyzing the surface quality of the printed model samples. For 9EA@NSS‐50, when the printing pressure was set to 0.59 MPa, the ink could be successfully extruded and printed to form a regular flat pattern. However, when the printing pressure was set to 0.48 Mpa, extrusion of the ink could also be achieved but the printing spacing became wider because of the small number of samples, which affected the smoothness of the printing material. When the sample volume in the syringe decreased, and the pressure was still 0.48 MPa during the later printing stage, the wire would break significantly impacting the quality of the printed samples (Supporting Information S1: Figure S23a). Secondly, the printing resolution and printing difficulty of the printed sample would be affected by the diameter of the needle. When the needle diameter is 0.2 mm, the printing resolution and exquisiteness of the printed sample could be enhanced, whereas simultaneously, the amount of p‐crystals in the ink was restricted. For example, the needle would become clogged when printing 9EA@NSS‐50 and cannot be printed normally. When the needle diameter was 0.5 mm, despite the slightly lower printing resolution of the sample, we could still incorporate more p‐crystals and print quickly without any obstacles (Supporting Information S1: Figure S23b). In addition, when the printing speed was set as 40 mm s^−1^, filament breakage would occur due to the printing speed being too fast, but when the printing speed was set as 20 mm s^−1^, this problem could be avoided (Supporting Information S1: Figure S23c). Finally, the final thickness and appearance of the printed material could be affected by the number of printed layers, and multi‐layer printing could increase the overall thickness of the sample to create a more complex printing structure (Supporting Information S1: Figure S23d).

The quality of the 9EA@NSS actuators was evaluated using stress−strain tests and SEM (Figure 3b–d). We explored the effect of different p‐crystal contents on the system's mechanical properties. The results showed that compared with the original NSS, the ultimate strain of the actuators decreased, indicating a reduction in the elasticity of the doped materials. However, the ultimate stress and Young's modulus of the actuators are improved (while they do not change regularly, which might be attributed to the fact that the doped p‐crystals were still relatively small compared with the matrix NSS), indicating that the doped materials had obtained more substantial rigidity. Stress‐strain experiments showed that all actuators possess good mechanical properties and could be stretched to 4–8 times of their original length. For example, the ultimate stress and strain at the break of the 9EA@NSS‐50 were 1.1 MPa and 477%, respectively (Figure 3d, Supporting Information S1: Figure S24). As a demonstration, 9EA@NSS‐50 (a 20 × 5 mm strip) could lift a 200 g steel object (Figure 3b). Scanning electron microscopy (SEM) images revealed that all crystals were uniformly dispersed into the NSS matrix to form the hybrid materials (Figure 3c). Overall, the printed actuators possess good mechanical properties, potentially benefiting their actuation performance.

### 2.2 Photomechanical actuators and photovoltaic performance of p‐crystals@NSS

**FIGURE 4:**
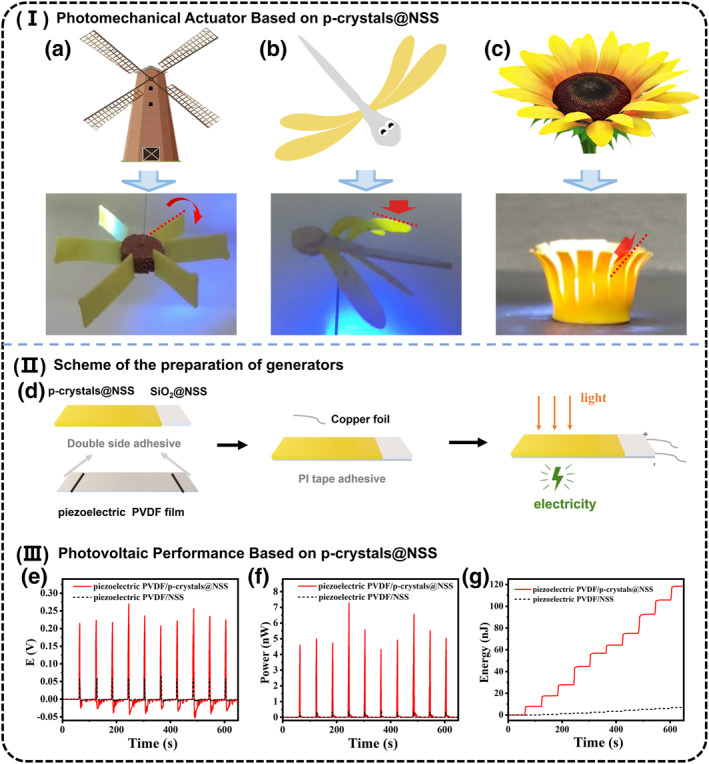
Photomechanical actuators and photovoltaic generators of 9EA@NSS‐50. I Photomechanical actuator based on 9EA@NSS‐50. (a) Photographs of a windmill robot rotated under the stimulation of 395 nm visible light; (b) a dragonfly flapped its wings in response to unilateral 395 nm visible‐light stimulation; (c) sunflower perform the flowering behavior when illuminated by 395 nm visible light. II Scheme of the preparation of generators. (d). III Photovoltaic performance based on 9EA@NSS‐50. (e) The voltage output of the bilayer generator under light stimuli over 10 cycles when loaded with a 10 MΩ resistor. (f) The instantaneous power output of piezoelectric PVDF/9EA@NSS‐50 generators under light stimulation. (g) The instantaneous energy output of piezoelectric PVDF/9EA@NSS‐50 generators under light stimulation. NSS, neutral silicone sealant.

The photomechanical response ability of p‐crystals@NSS could be applied to the preparation of various photomechanical‐responsive robots. Furthermore, robots with more complex and exquisite structures could be obtained through 4D printing technology. As a concept demonstration, a windmill with six fan blades consisting of two materials was printed, and colored NSS without p‐crystals was printed in the middle, whereas the fan blade portion was printed with the 9EA@NSS‐50. When irradiated with unilateral 395 nm visible light in the vertical direction, the blade would drive the whole windmill to rotate. Additionally, we printed a tiny dragonfly made of two materials, whose wings were printed with 9EA@NSS‐50, while its body portion was printed with SiO_2_@NSS‐50. The dragonfly could flap its wings in response to unilateral 395 nm visible‐light stimulation. Furthermore, we printed a sunflower that can perform the flowering behavior when illuminated by 395 nm visible light (Figure 4a–c and Video [Link], [Link], [Link]).

The excellent photomechanical capability of p‐crystals@NSS also indicated its potential to be used in the manufacture of energy conversion devices to convert photon energy into electricity. Therefore, we coupled the 9EA@NSS‐50 actuator with a piezoelectric PVDF film to fabricate a bilayer generator (Figure 4d, Supporting Information S1: Figure S25). As shown in Supporting Information S1: Figure S26, when the bilayer generator was irradiated by the unilateral 395 nm UV‐light in the vertical direction, the 9EA@NSS‐50 actuator would bend away from light, thus driving the deformation of the piezoelectric PVDF film to generate electricity. Once the light was removed, this bilayer generator returned to its original state. The power output was related to the bending speed and amplitude. In comparison without an actuator, the piezoelectric PVDF film with SiO_2_@NSS‐50 had only a weak signal output of about 1/5 of the 9EA@NSS‐50 signal output. This energy conversion could be recycled at least 10 times. As a result, the energy accumulated in the resistor in 14 min outputting a peak voltage reached 0.27 V when a 10 MΩ resistor was loaded, with the instantaneous peak power of 7.28 nW and power density of 79.65 μW kg^−1^, which is comparable to state‐of‐the‐art generators fabricated using crystalline materials (Figure 4e–g). This work highlights the potential of using p‐crystals to generate clean energy and merits more efforts in the future.

In summary, we successfully innovated a simple, general 4D printed approach to fabricate photomechanical actuators using crystalline smart materials. The inexpensive and accessible NSS was first chosen as the photomechanical 4D printing matrix and uniformly doped with nine types of photoresponsive crystalline materials, including acylhydrazone‐, anthracene‐, olefin‐, and azobenzene‐based molecular crystals and COFs. Notably, it is the first time to use COFs to fabricate photo‐responsive actuators. The formed hybrid materials exhibited excellent photomechanical performance. We systematically investigated the effects of different printing conditions, different types of photoresponsive crystalline materials, and the doping amount of crystals on the performance of the actuators. It was found that 9EA@NSS‐50 had the best photomechanical performance, and the bending angle can reach *θ* = 20.98° after illumination for 3 s. Taking advantage of the molding capabilities of 4D printing, small windmills, dragonflies, and sunflowers with alternating dual materials that can perform behaviors such as rotation (the first for photomechanical crystalline material), wing flapping, and blossom were printed. Furthermore, coupling with a piezoelectric material, the actuator could also periodically output electricity of 0.27 V with a 10 MΩ resistor loaded upon light irradiation. This study paves a new avenue for fabricating photomechanical actuators and electricity generators and probably provides a new potential path to furnish energy for drones in the future, which broadens the application scope of crystalline smart materials, especially COFs.

## 3 METHODS

### 3.1 Synthesis of p‐crystals

(*E*)‐4‐chloro‐*N'*‐(4‐methoxybenzylidene)benzohydrazide(Ac‐1h),9‐((Ethylimino)methyl) anthracene(9EA), (*Z*)‐2‐(3,5‐bis(trifluoromethyl)phenyl)‐3‐(naphthalen‐2‐yl) acrylonitrile‐*α* (BNA), (Z)2‐(3,5‐bis(trifluoromethyl)phenyl)‐3‐(naphthalen‐2‐yl) acrylonitrile (BOV1N), NKCOF‐15, VCOF‐1 was synthesized according to literature. (*E*)‐4‐chloro‐*N'*‐(thiophen‐2‐ylmethylene)benzohydrazide(Ac‐a) was synthesized using a method similar to Ac‐1h: Thiophene‐2‐carbaldehyde (0.561 g, 5.0 mmol, 1.0 equivalent) was added to 4‐chlorobenzohydrazide (0.853 g, 5.0 mmol, 1.0 equivalent) in ethanol (50 mL) and refluxed for 3‒4 h with the addition of a catalytic amount of glacial acetic acid. Upon completion of the reaction monitored by TLC, the obtained precipitates were filtered off and recrystallized from ethanol. Finally, upon slow evaporation from the acetonitrile solution, needle‐like Ac‐a crystals were generated. Commercial 9‐anthraldehyde (9AA), (*E*)‐4‐(phenyldiazenyl) aniline (Azo‐1) were directly used without further purification to grow crystals. CH_2_Cl_2_ was added to a powder of 9EA (1.0 g) until completely dissolved to obtain a saturated solution, which was evaporated at room temperature for 1 day to yield yellow needle‐like crystals. Commercial 9AA was directly used without further purification to grow crystals via a similar procedure as 9EA. Ethanol as a poor solvent was injected into the dichloromethane solution of BNA, BOV1N to achieve a nearly saturated system, and then the needle‐like crystals (BNA) quickly nucleated and precipitated. Acetonitrile was added to a powder of Ac‐1h, Ac‐a, and Azo‐1 (1.0 g) until completely dissolved to obtain a saturated solution, which was evaporated at room temperature for 3 days to form crystals. Among these crystals, 9EA, 9AA, BNA, BOV1N, Ac‐1h and Ac‐a formed needle‐like crystals, Azo‐1 formed sheet‐like crystals, and NKCOF‐15, VCOF‐1 formed microcrystalline powders. 9EA(CCDC#1982847), 9AA(CCDC#1510568), BNA(CCDC#2032920), BOV1N(CCDC#1904295), Ac‐1h(CCDC#1861513), Azo‐1 (CCDC#761536), NKCOF‐15, and VCOF‐1 possessed the reported crystal structures, while Ac‐a (CCDC#2356358) generated a new structure.

[CCDC 1982847, 1510568, 2032920, 1904295, 1861513, 761536, 2356358 contain the supplementary crystallographic data for this paper. These data can be obtained free of charge from The Cambridge Crystallographic Data Centre via www.ccdc.cam.ac.uk/data_request/cif.].

### 3.2 Synthesis of p‐crystal@NSS precursor inks

P‐crystals were pre‐milled into finer crystal powders (440 rpm, 3.5 h), respectively, weighing the corresponding mass of p‐crystals and NSS, and the two materials were fully mixed at room temperature, forming a mixture of preparative printing materials with molar ratios of NSS and p‐crystals of 300:1, 100:1, 75:1, 50:1 and 25:1, recorded as p‐crystal @ NSS‐300, p‐crystal @ NSS ‐100, p‐crystal @ NSS ‐75, p‐crystal @ NSS ‐50, and p‐crystal‐25 @ NSS. The light‐responsive material was loaded into the 3D printing cylinder, and the bubbles in the printing material were removed by centrifugation (5500 rpm, 10 min) to prevent breakage during the printing process.

### 3.3 4D printing of the p‐crystal@NSS inks

For p‐crystal @ NSS ‐50, the diameter of the chosen conical needle tip was 0.5 mm. With an extrusion pressure of 0.59 MPa by an air‐powered dispensing system, the moving speed was 20 mm s^−1^, the printing interval was 0.4 mm, the wire closing time in advance was 200 ms, and the broken wire lifting distance was 1 mm. After being naturally dried for 48 h, the printed material could be easily peeled off from the substrate.

### 3.4 4D printing of the SiO2@NSS inks

For SiO_2_@NSS ‐50, the diameter of the chosen conical needle tip was 0.5 mm. With an extrusion pressure of 0.59 MPa by an air‐powered dispensing system, the moving speed was 30 mm s^−1^, the printing interval was 0.45 mm, the wire closing time in advance was 200 ms, and the broken wire lifting distance was 1 mm. After being naturally dried for 48 h, the printed material could be easily peeled off from the substrate.

### 3.5 The preparation of generators

A commercial piezoelectric PVDF film measuring 23 μm with an aluminum electrode on both sides was acquired from Jinzhou KEXIN Electronic Material Co., Ltd. It was cut into the strips (25 × 5 mm). First, ensure that the upper and lower aluminum electrodes do not touch during operation by adhering an insulating PI tape on one side of the piezoelectric PVDF film. In a typical formulation, the p‐crystals@NSS Inks were 4D printed in strips (20 × 5 mm) on the PVDF film. Then, copper foils were attached on both sides of PVDF by PI tape as electrodes to connect with the test instrument.

## AUTHOR CONTRIBUTIONS

Z. Z conceived and designed the project. Y. L. and J. L. performed the experiments. L. H. E. L. and P. C. helped with the structural characterization analysis. J. W., T. W. and S. G. helped to analyze the results of photovoltaic tests. Z. Z. and Y. L. wrote the manuscript with contributions from all the authors.

## CONFLICT OF INTEREST STATEMENT

The authors declare no conflicts of interest.

## ETHICS STATEMENT

No animal or human experiments were involved in this study.

## Supporting information

Supporting Information S1

**Video S1:** The reversible bending process of 9EA@NSS‐50 under visible light stimulation.

**Video S2:** Windmill robot actuator based on 9EA@NSS‐50.

**Video S3:** Dragonfly robot actuator based on 9EA@NSS‐50.

**Video S4:** Sunflower actuator based on 9EA@NSS‐50.

## Data Availability

All data supporting the findings of this study are available within the article as well as the Supplementary Information file or available from the corresponding authors on reasonable request.
