# Supplementary material for: Incorporating crystalline smart materials to fabricate 4D printed photomechanical actuators with photovoltaic performance
Source: Smart Mol. 2025 Nov 24;4(1):e70026. doi: 10.1002/smo2.70026 (PMC13104073; doi:10.1002/smo2.70026)
Supplement: Supplementary file 1 — Supporting Information S1 [file SMO2-4-e70026-s001.doc]

**Supplementary information**

**Incorporating Crystalline Smart Materials to Fabricate 4D Printed Photomechanical Actuators with Photovoltaic Performance**

*Yujie Liu,^1^ Jinjin Liu,^1^ Liqin Hao,^1^ En Lin,^1^ Jiaxi Wang,^1^ Tonghai Wang**,^1^ Shubo Geng,^1^ Peng Cheng^1,2,3^ and Zhenjie Zhang^*,1,2,3^*

[1] Y. Liu, J. Liu, L. Hao, E. Lin, J. Wang, T. Wang, S. Geng, Prof. P. Cheng, Prof. Z. Zhang

College of Chemistry, State Key Laboratory of Medicinal Chemical Biology, Nankai University, 300071 Tianjin, China

[2] Prof. P. Cheng, Prof. Z. Zhang

Frontiers Science Centre for New Organic Matter, Key Laboratory of Advanced Energy Material Chemistry (Ministry of Education), Nankai University, 300071 Tianjin, China

[3] Prof. Z. Zhang

Nankai International Advanced Research Institute (Shenzhen Futian), Nankai University, 300071 Tianjin, China

*Corresponding Author: Zhenjie Zhang, zhangzhenjie@nankai.edu.cn.

Table of Contents

**SI-1. Details on experimental methods**

**SI-2. Supplementary figures**

**SI-3. Supplementary tables**

**SI-1. Details on experimental methods**

**General information**

Chemicals and solvents were purchased from commercial sources and used as received without further purification.

**Scanning Electron Microscopy:** Samples were transferred to conductive carbon tape on a sample holder disk and coated by Au-sputter for 3 min. Hitachi SU3500 SEM instrument was used to acquire images using a 30 kV energy source under vacuum.

**Powder X-ray Diffraction Analysis:** PXRD data were collected at ambient temperature on Rigaku dmax 2500 diffractometer using Cu K-α radiation, with a scan speed of 1 sec/step, 2θ ranging from 2º to 40º. For all samples, the experimental backgrounds were not corrected.

**Fourier Transform Infrared Spectroscopy:** FT-IR spectra of preparative samples were recorded using a Nicolet iS 50 ATR-FT-IR instrument.

**Single Crystal X-ray diffraction:** SCXRD data of **Ac-a** were collected at 120 K via an Oxford Cryo stream system on a SuperNova (Mo) X-ray Source with a micro-focus sealed X-ray tube. A single crystal was doped into Paratone-N oil, then mounted via a loop, and quickly transferred to the diffractometer, and SCXRD data was collected at 120 K under N_2_ flow. The structure was solved and refined using Olex2 with 'XS' and 'XL' plug-in.

**Nuclear magnetic resonance spectroscopy (NMR):** ^1^H NMR spectra were recorded on Bruker AV400 instruments at 400 MHz. Chemical shifts were reported in parts per million (ppm) downfield from internal tetramethylsilane.

**LED lamp:** The light-responsive crystal and the 4D printing actuators were illuminated by LED ultraviolet lamps (365nm for BNA, BOV1N, Ac-1h, Ac-a, and Azo-1, I = 1300 mW cm^-2^. 395 nm for 9EA, 9AA, V-COF-1 and NKCOF-15 I = 3560 mW cm^-2^), and the distance between all lights and the illuminated sample was 2 cm. In the demonstration experiment of sunflower flowering, LED light of 395 nm 1300 mW cm^-2^ was used.

**SI-2. Supplementary figures**

**^1^H NMR data for crystals**


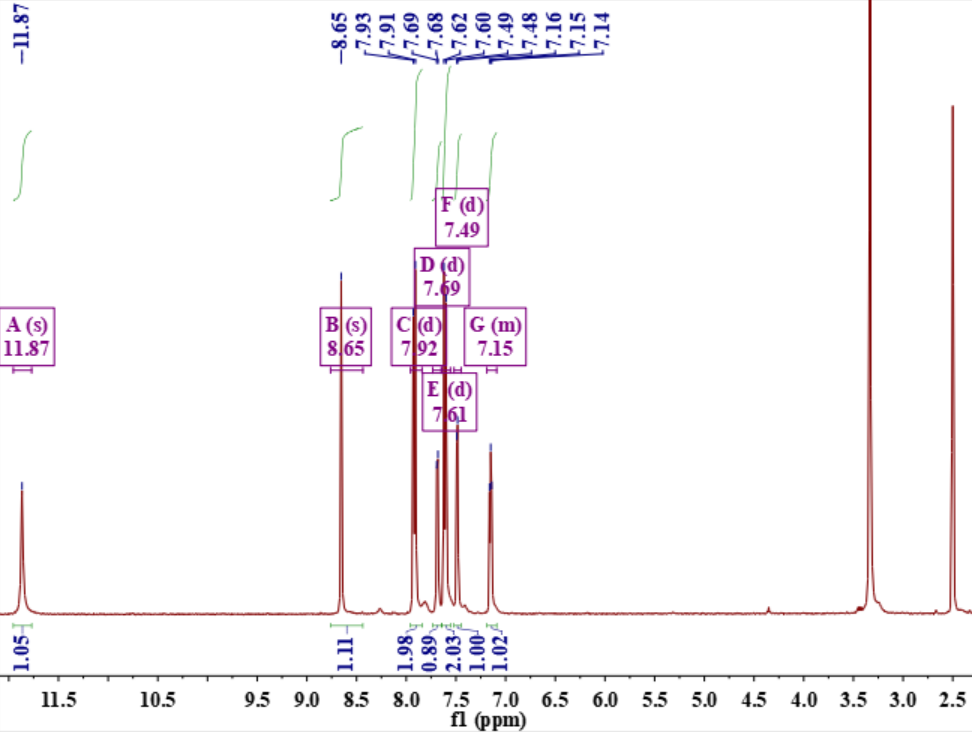


**Figure S1**. ^1^H NMR spectrum of **Ac-a**.


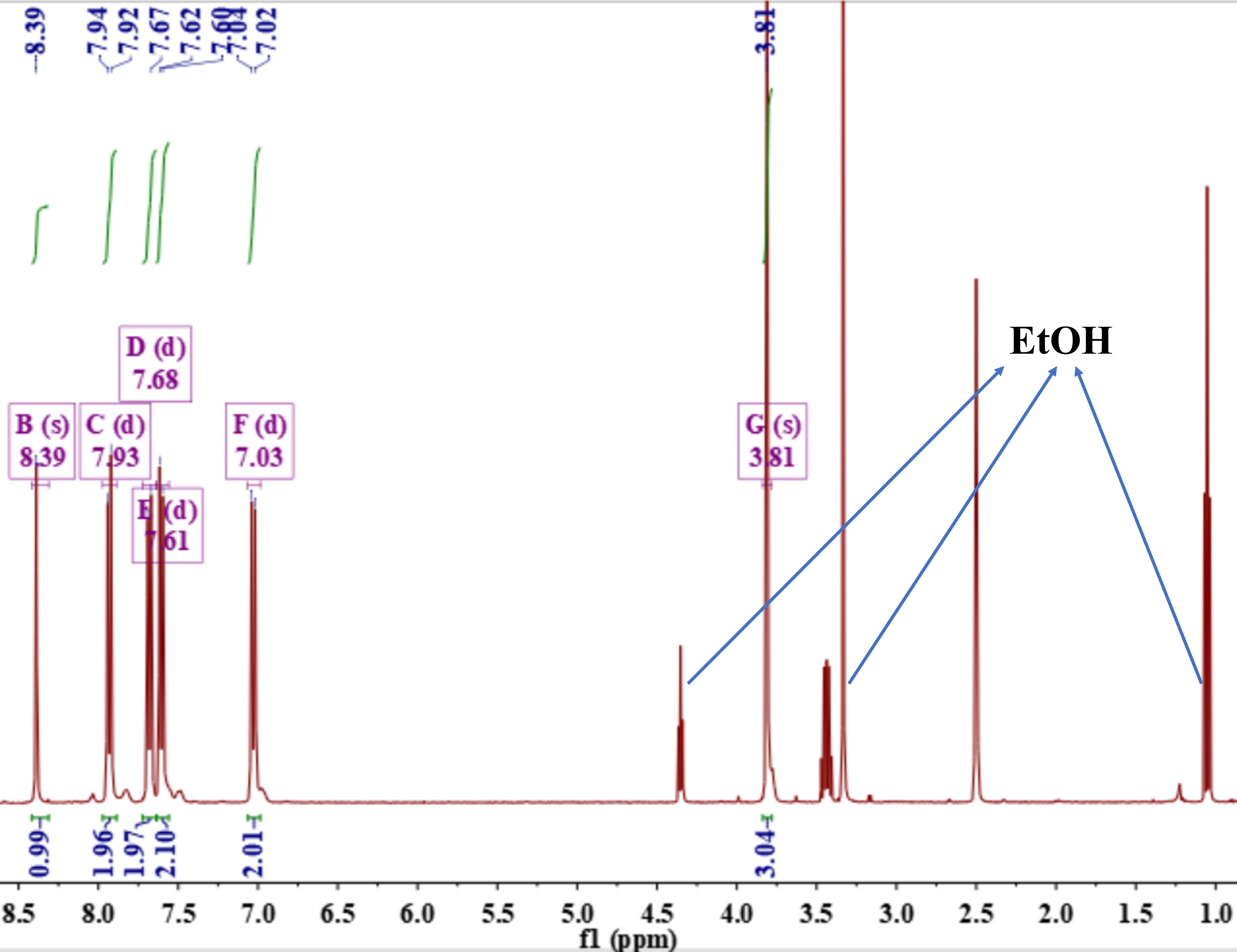


**Figure S2**. ^1^H NMR spectrum of **Ac-1h**.


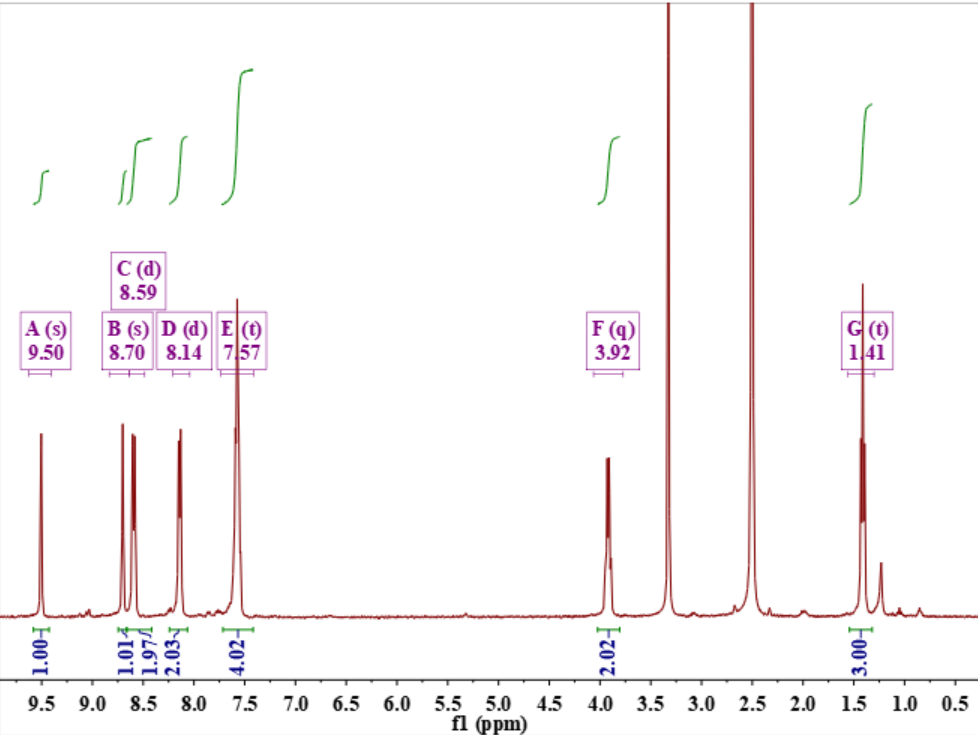


**Figure S3**. ^1^H NMR spectrum of **9EA**.


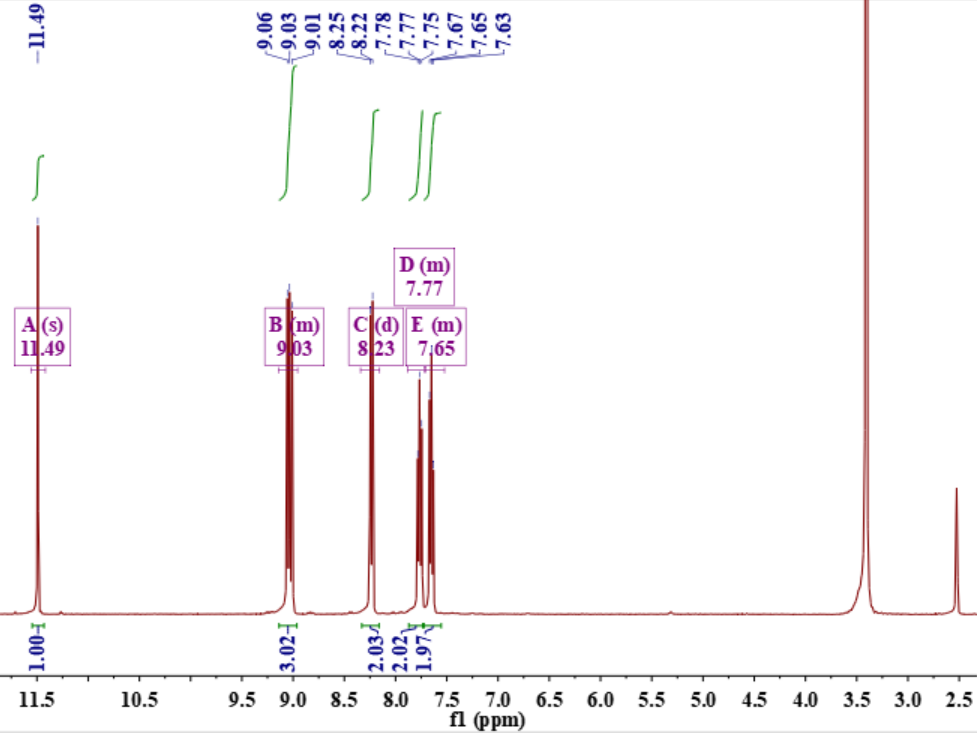


**Figure S4**. ^1^H NMR spectrum of **9AA**.


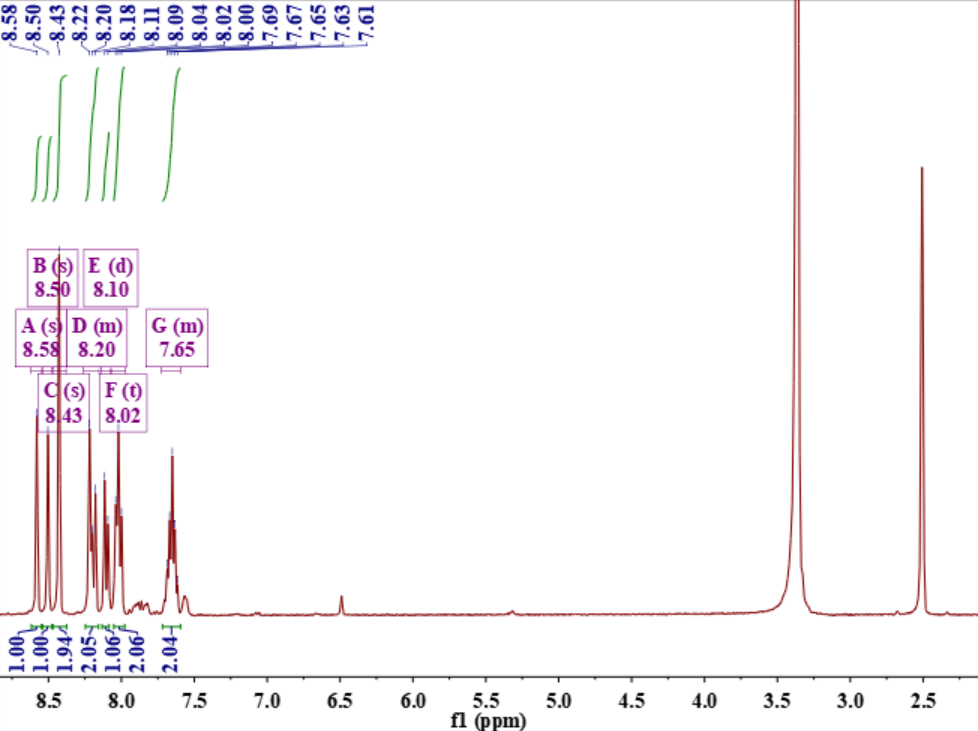


**Figure S5**. ^1^H NMR spectrum of **BNA**.


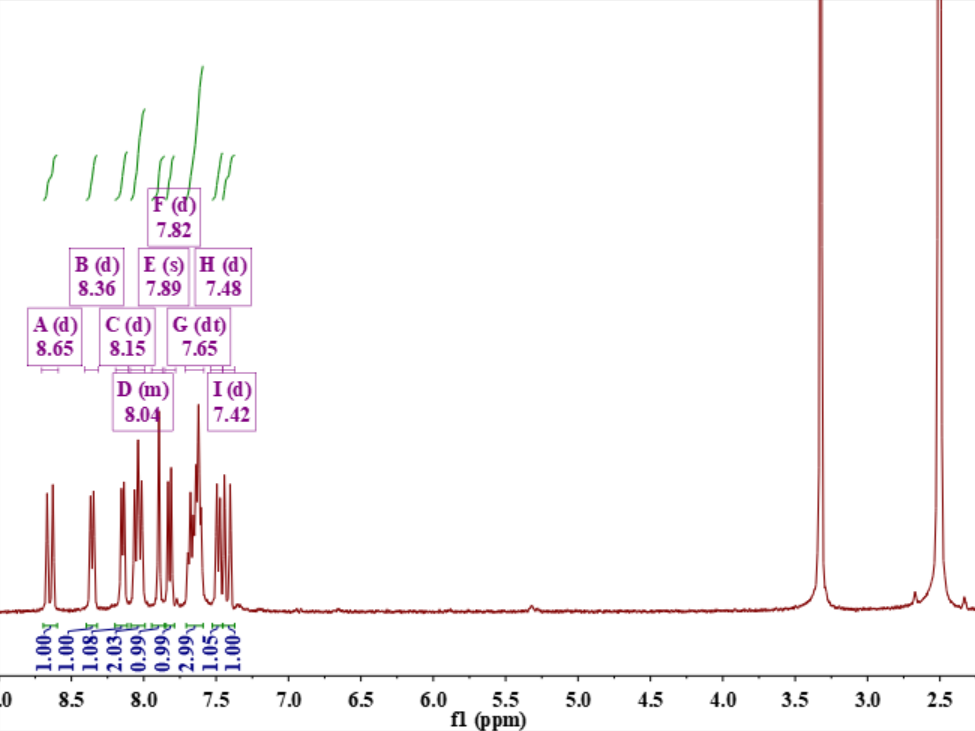


**Figure S6**. ^1^H NMR spectrum of **BOV1N**.


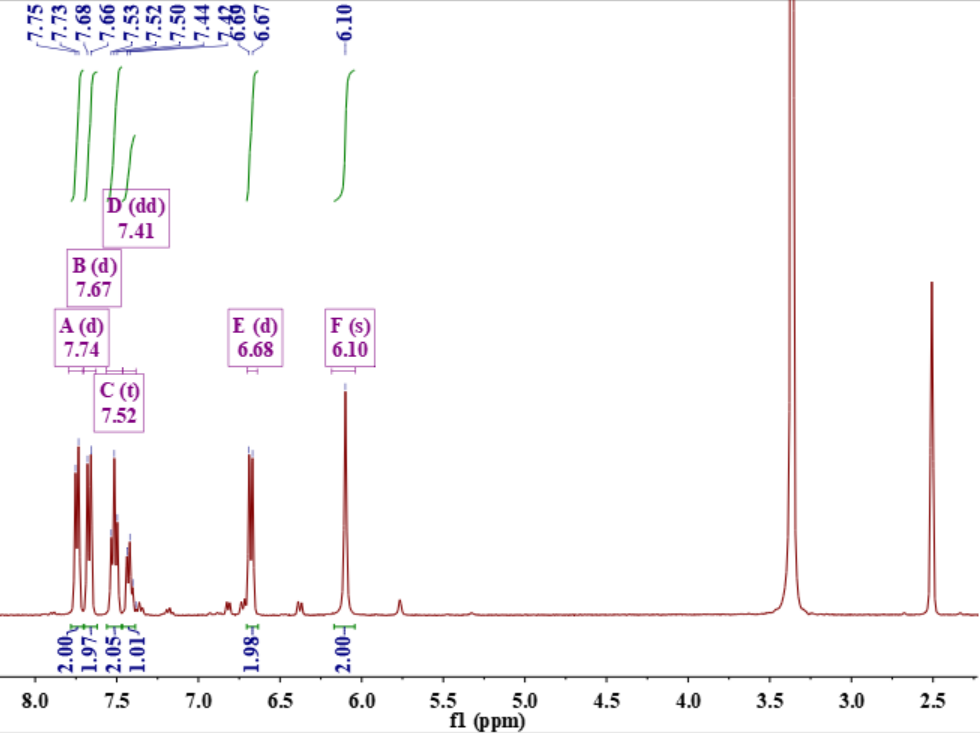


**Figure S7**. ^1^H NMR spectrum of **Azo-1**.


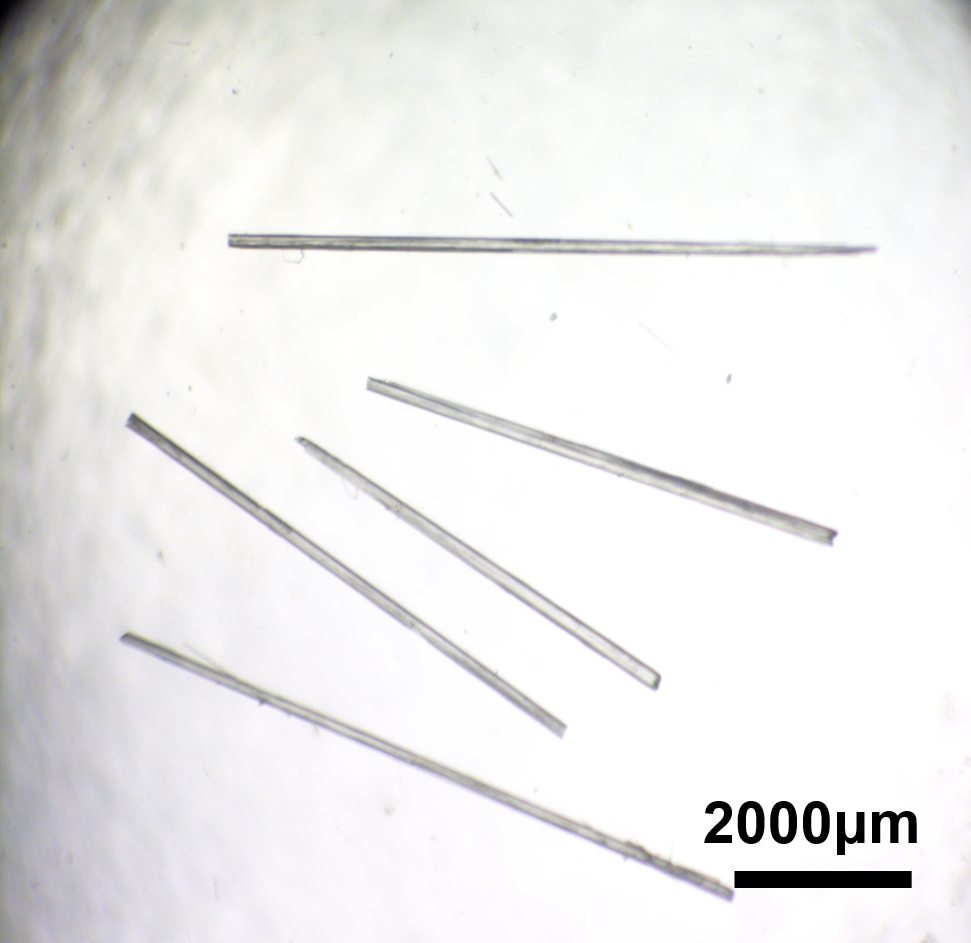


**Figure S8**. Crystal morphology of **Ac-a**.


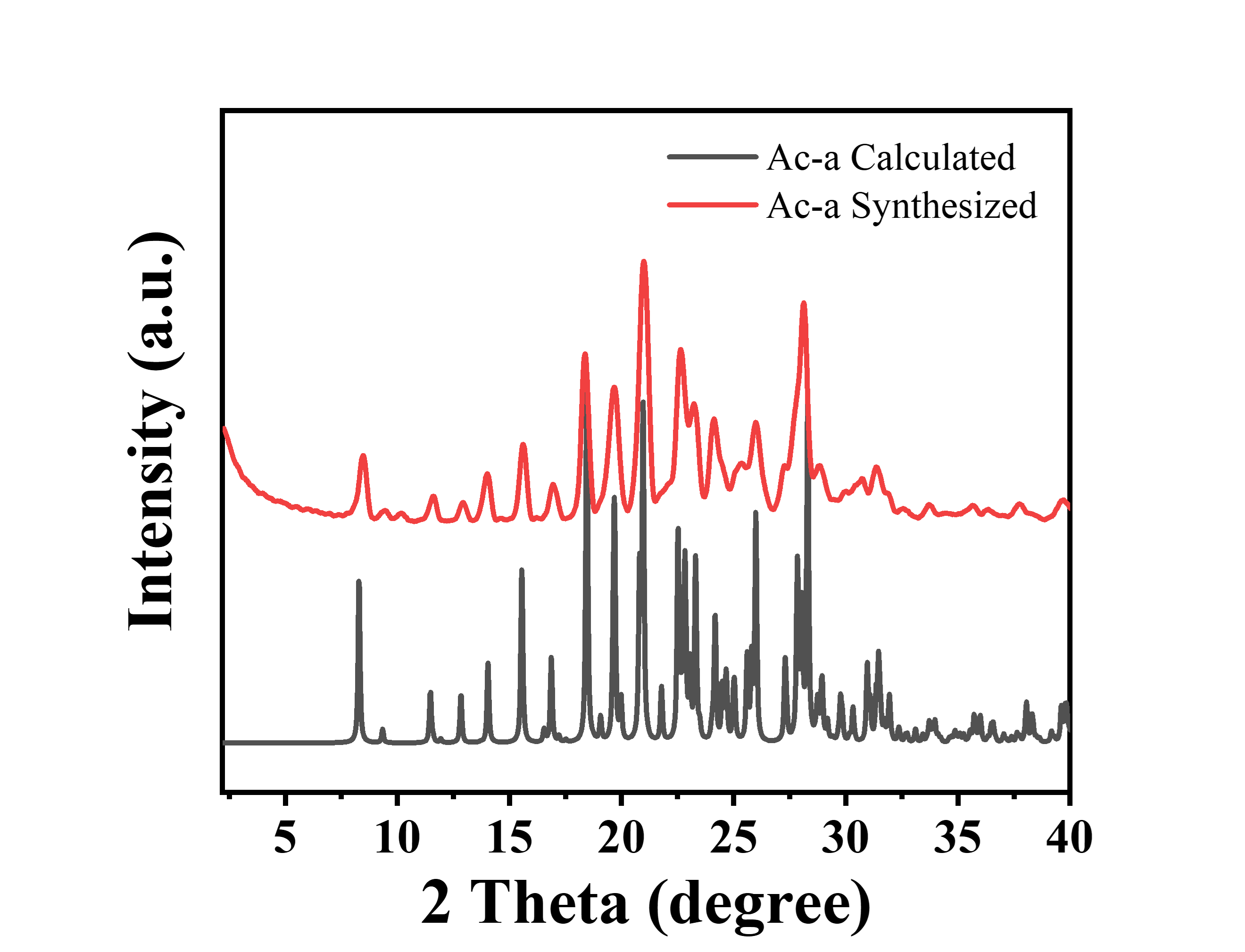


**Figure S9**. Calculated and as-synthesized PXRD patterns of **Ac-a**.


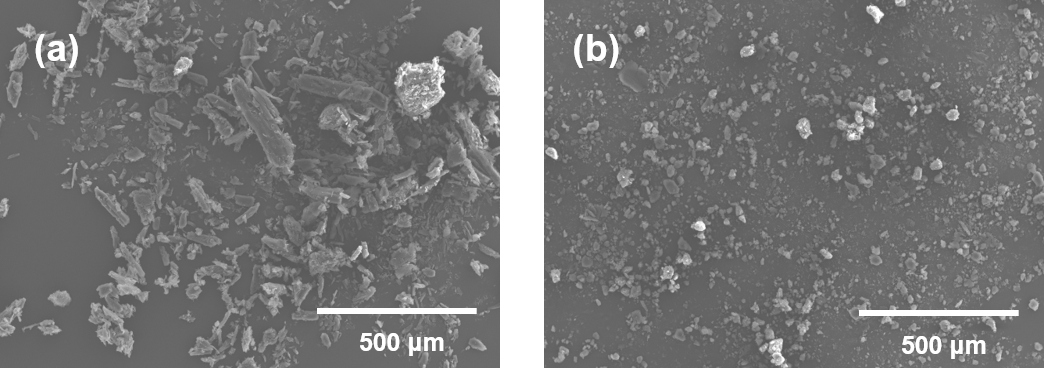


**Figure S10**. The SEM image of Ac-a crystal particles before and after high-speed ball milling.


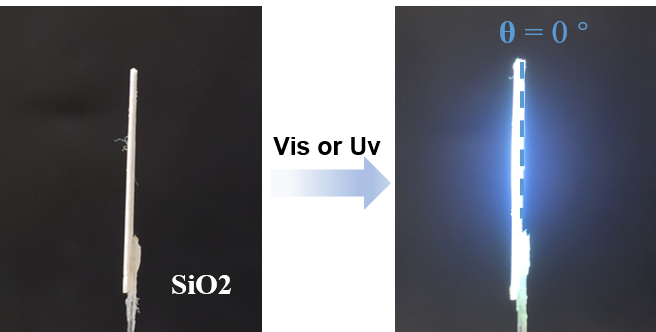


**Figure S11**. Comparison of photoresponsive bending behavior of **SiO_2_@NSS-50** (membrane length, 2.0 cm; width, 5.0 mm, thickness, 450.0 µm).


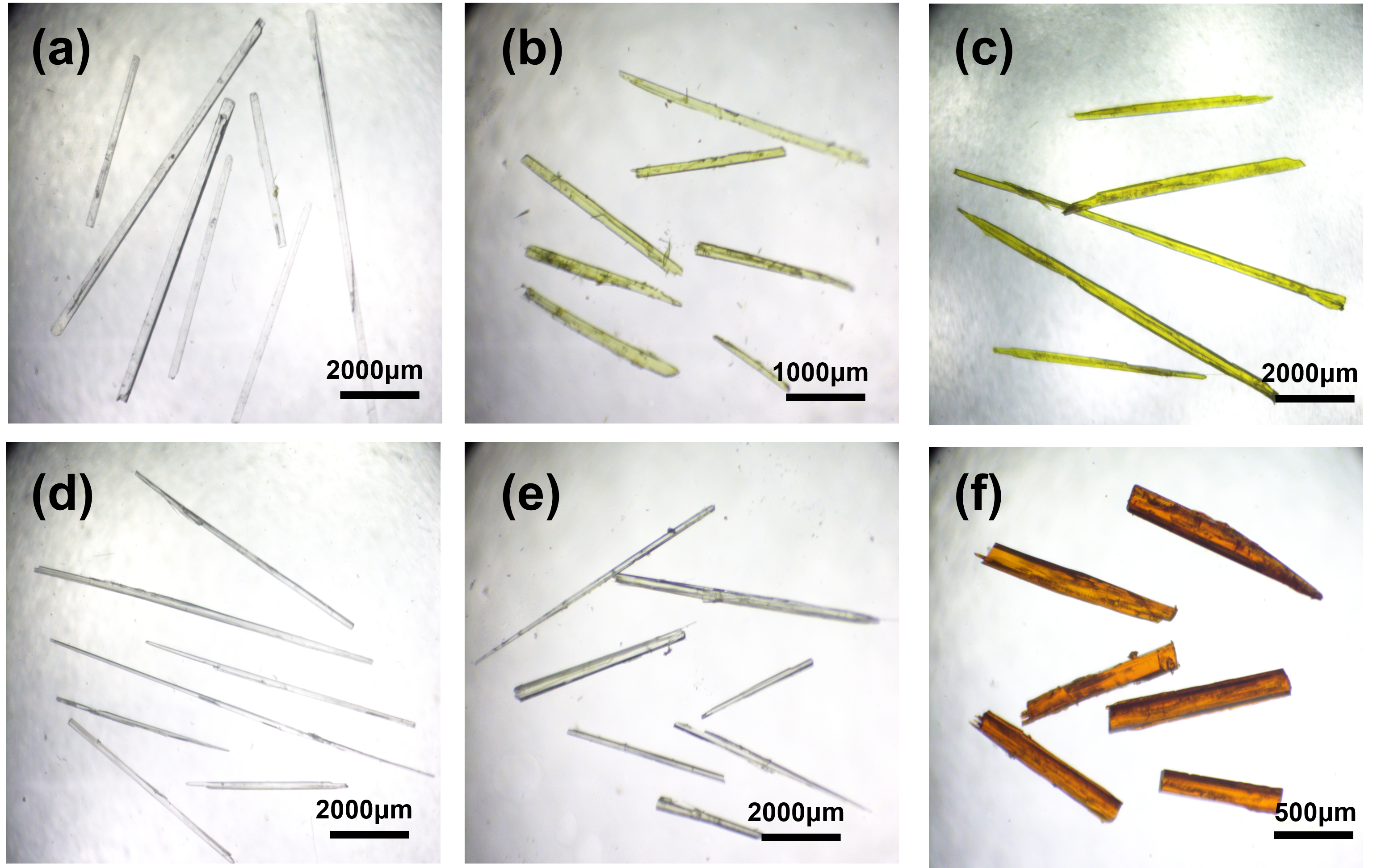


**Figure S12**. Crystal morphology of **Ac-1h** (a), **9EA** (b), **9AA** (c), **BNA** (d), **BOV1N** (e), and **Azo-1** (f).


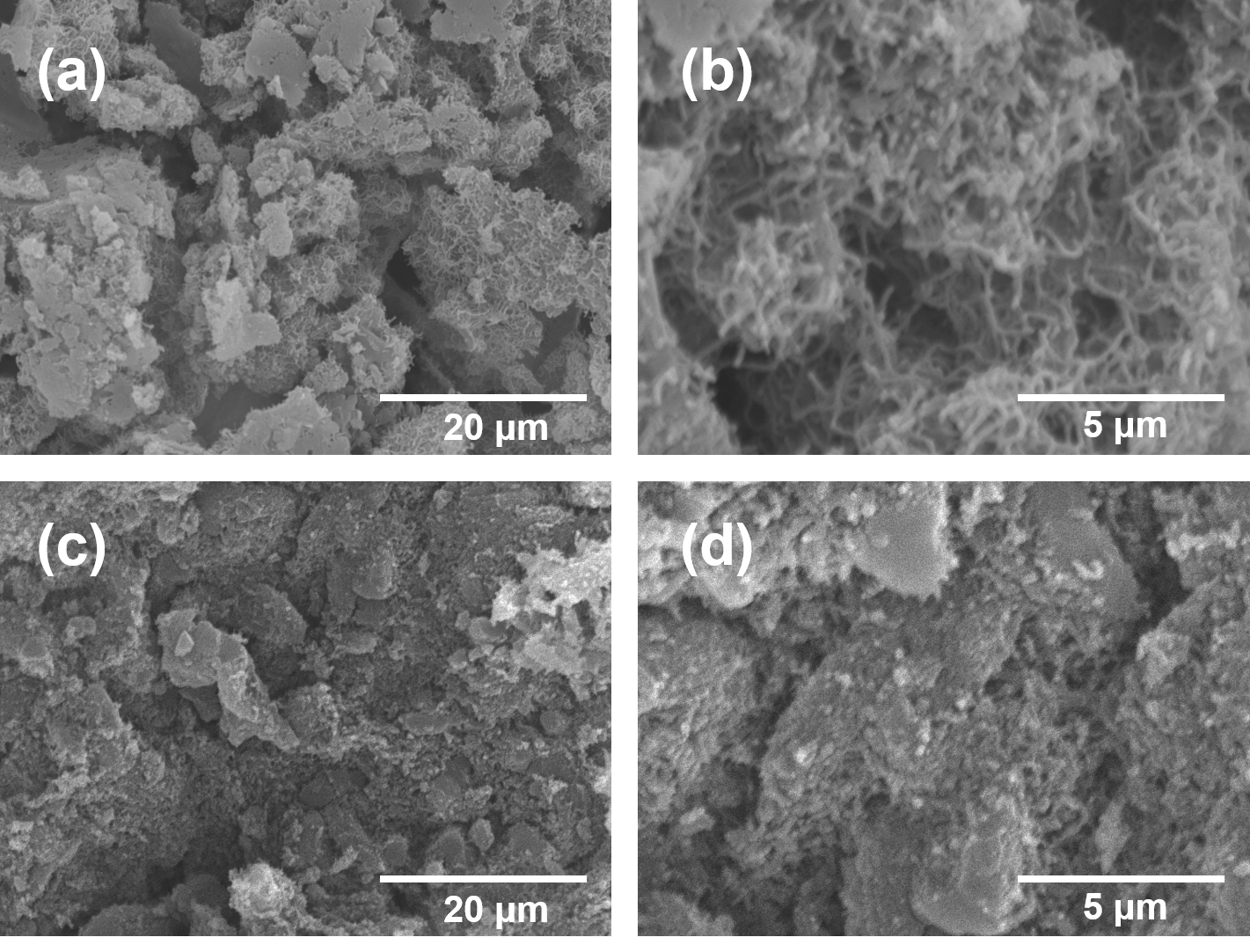


**Figure S13**. SEM image of **NKCOF-15**(a) (b), and **VCOF-1** (c) (d).


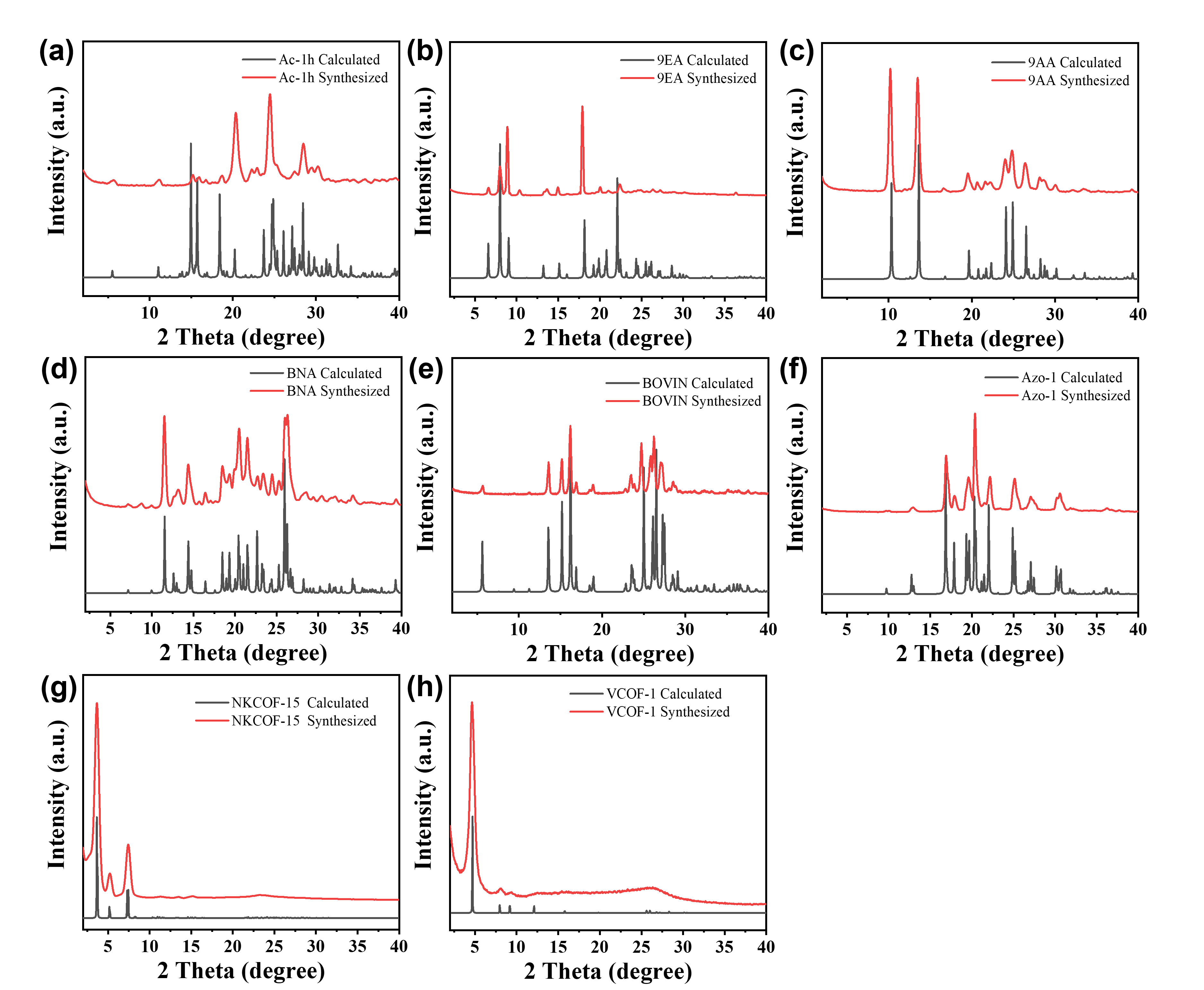


**Figure S14**. Calculated and as-synthesized PXRD patterns of **Ac-1h** (a), **9EA** (b), **9AA** (c), **BNA** (d), **BOV1N** (e), **Azo-1** (f), **NKCOF-15** (g) and **VCOF-1** (h).


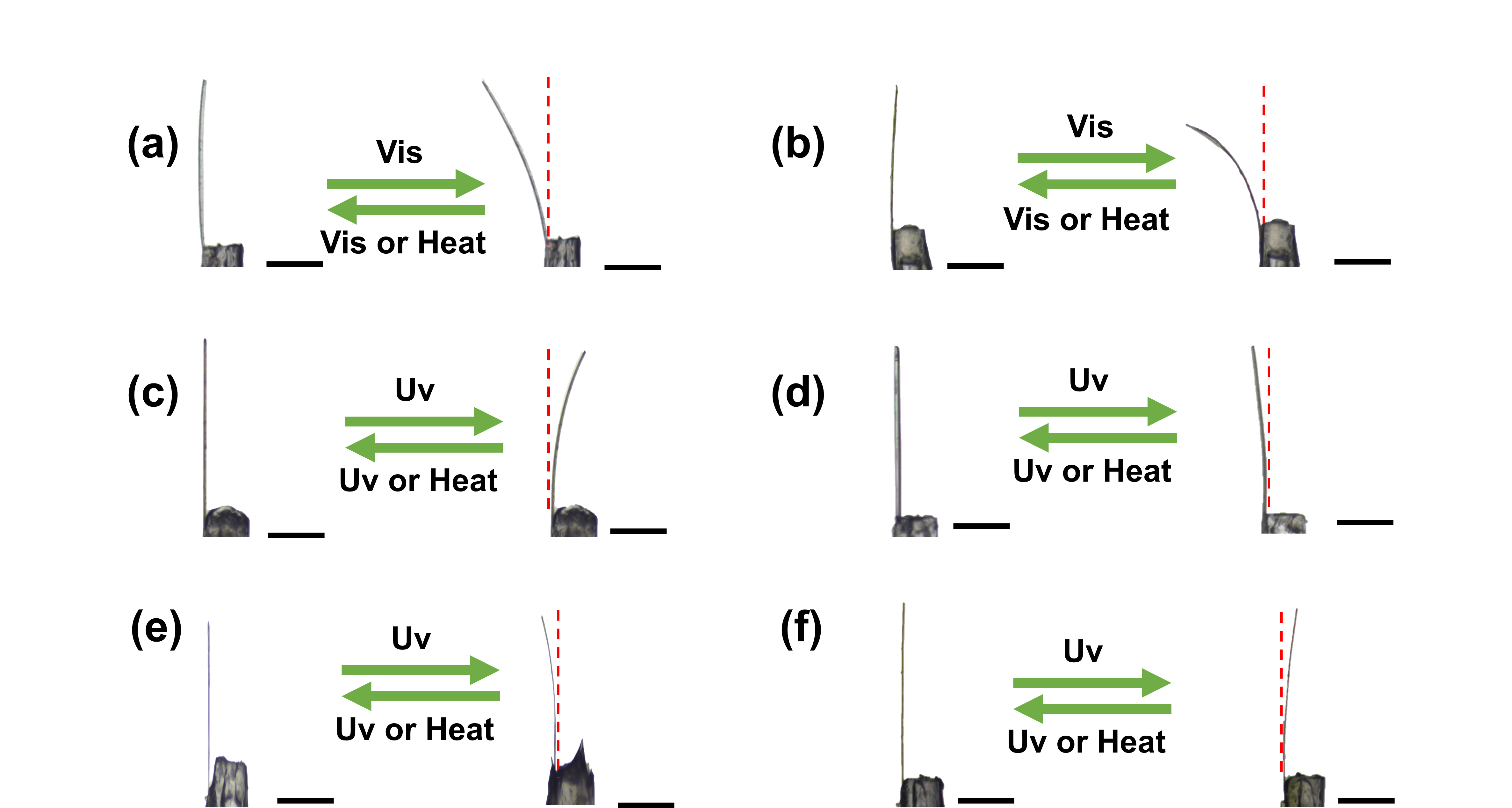


**Figure S15**. Photoresponsive bending performance of **Ac-1h** (a), **9EA** (b), **9AA** (c), **BNA** (d), **BOV1N** (e), and **Azo-1** (f) crystals triggered by light irradiation or heating (scale bar is 500 µm).


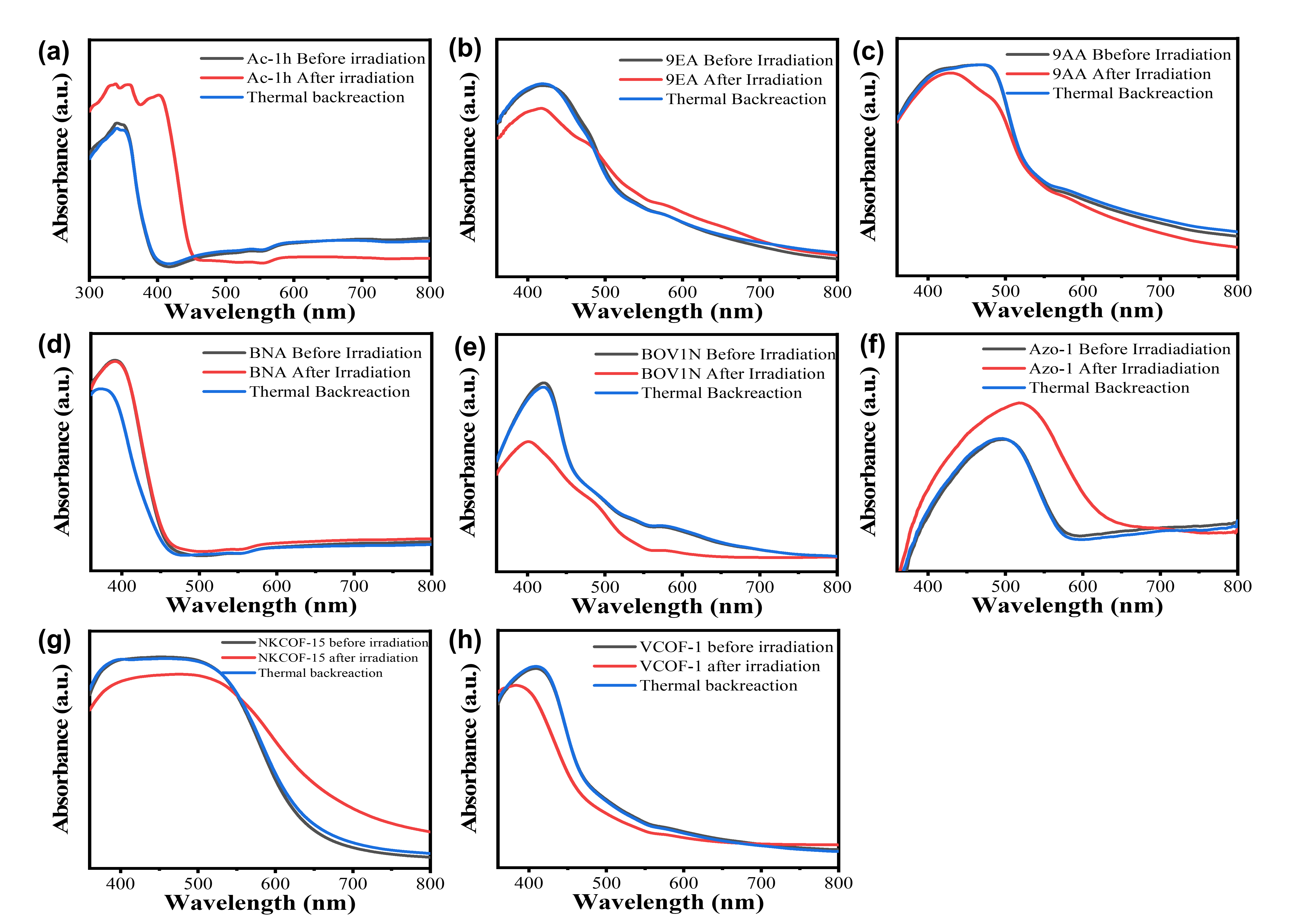


**Figure S16**. UV-Vis spectra of **Ac-1h** (a), **9EA** (b), **9AA** (c), **BNA** (d), **BOV1N** (e), **Azo-1** (f), **NKCOF-15** (g) and **VCOF-1** (h) before and after light irradiation and thermal backreaction or ambient backreaction.


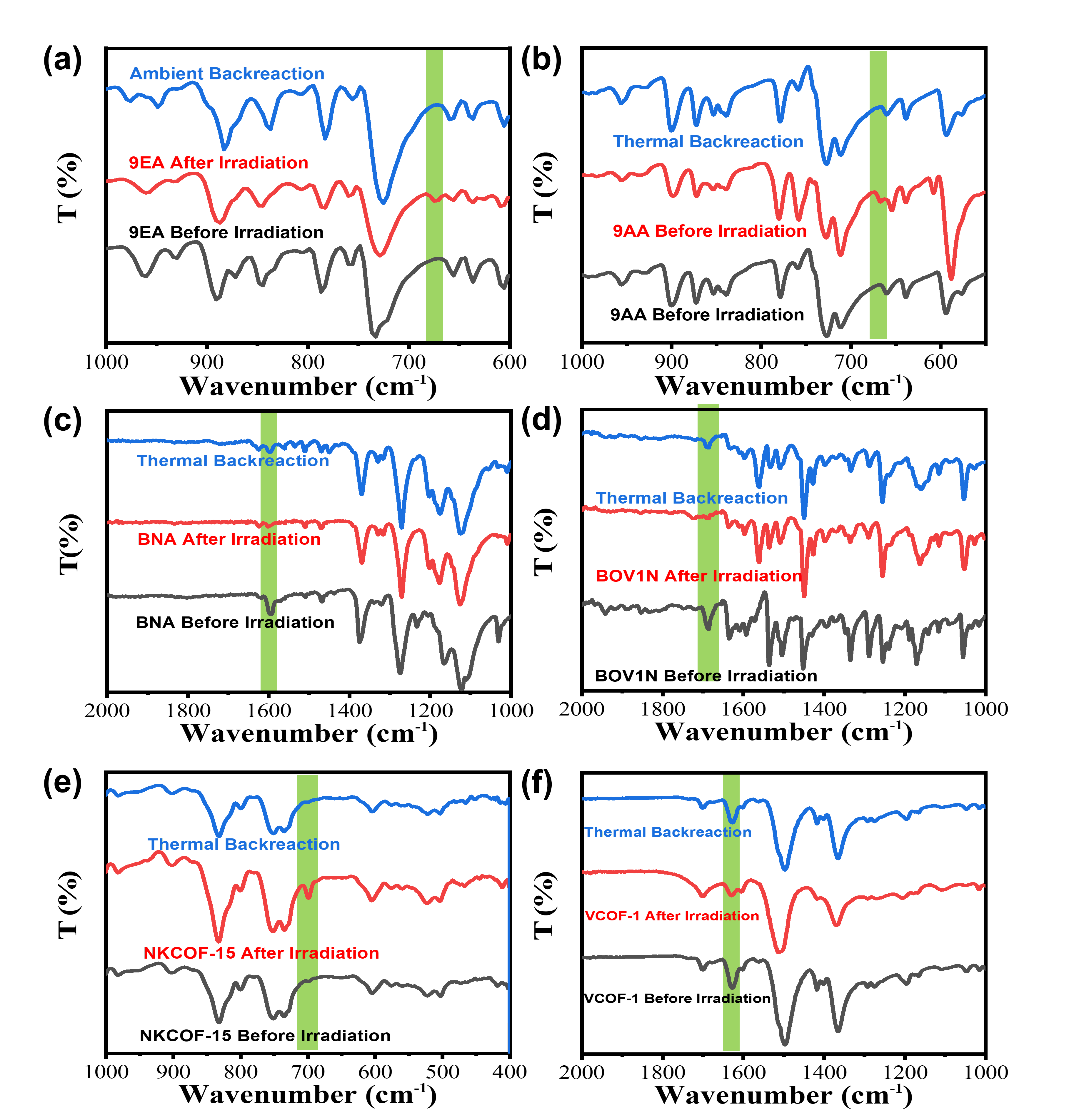


**Figure S17**. FT-IR spectra of **9EA** (a), **9AA** (b), **BNA** (c), **BOV1N** (d), **NKCOF-15** (e) and **VCOF-1** (f) before and after light irradiation and thermal backreaction or ambient backreaction.


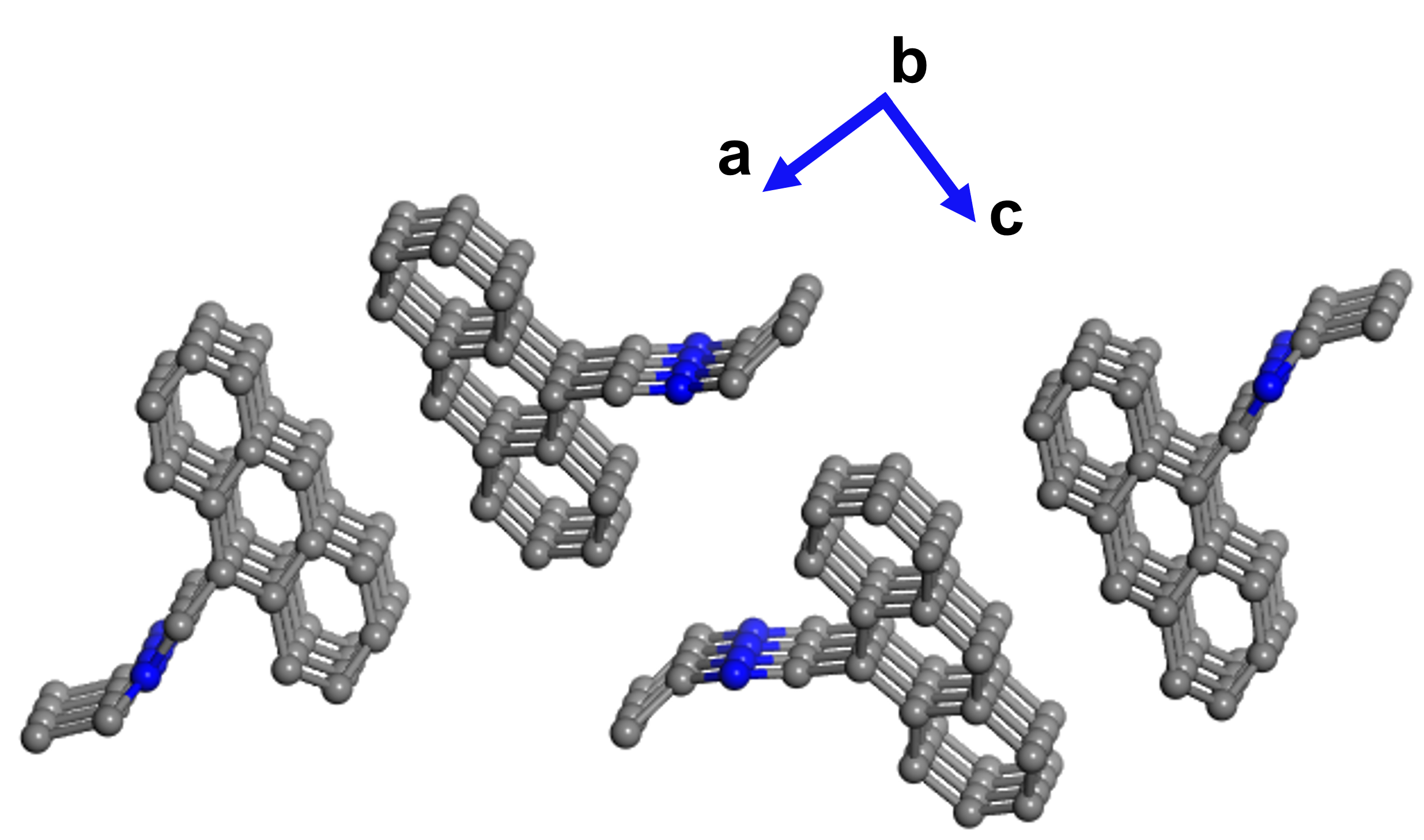


**Figure S18**. (a) Crystal structure of **9EA** packing along *b* direction (Vertical distance between adjacent anthracene is 4.23 Å) Carbon in gray, nitrogen in blue.


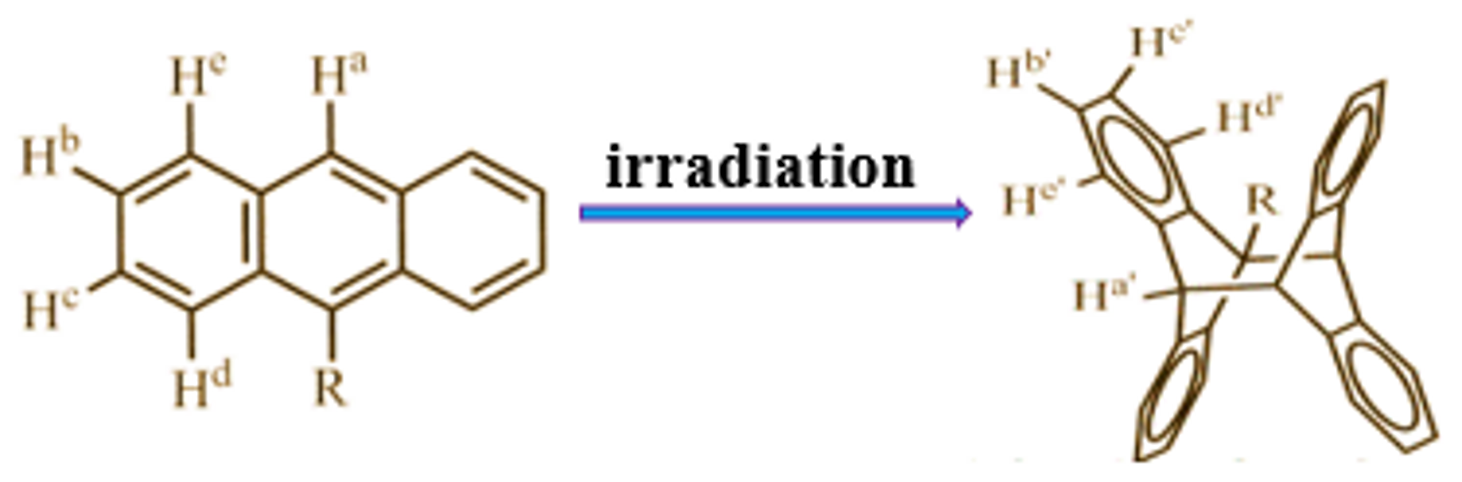

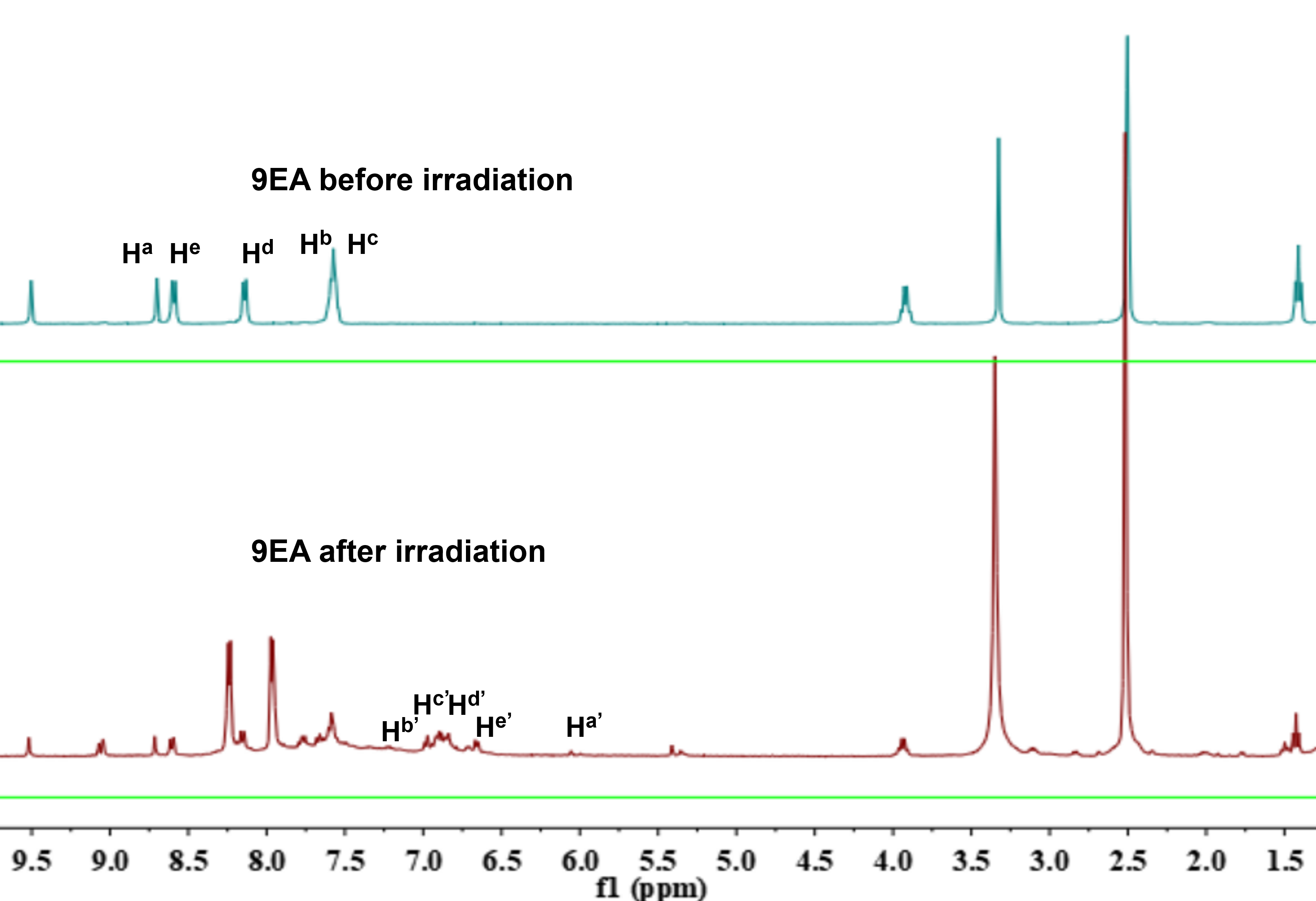


**Figure S19**. ^1^H NMR spectra of **9EA** before irradiation (top), after irradiation (bottom) with visible light for 3 days.


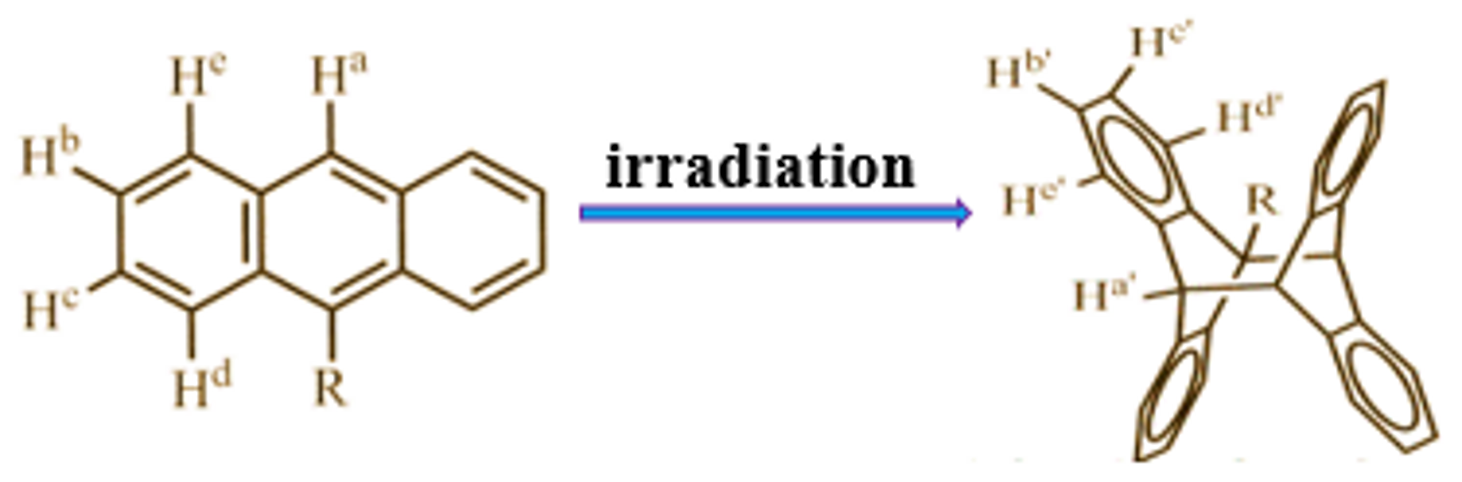


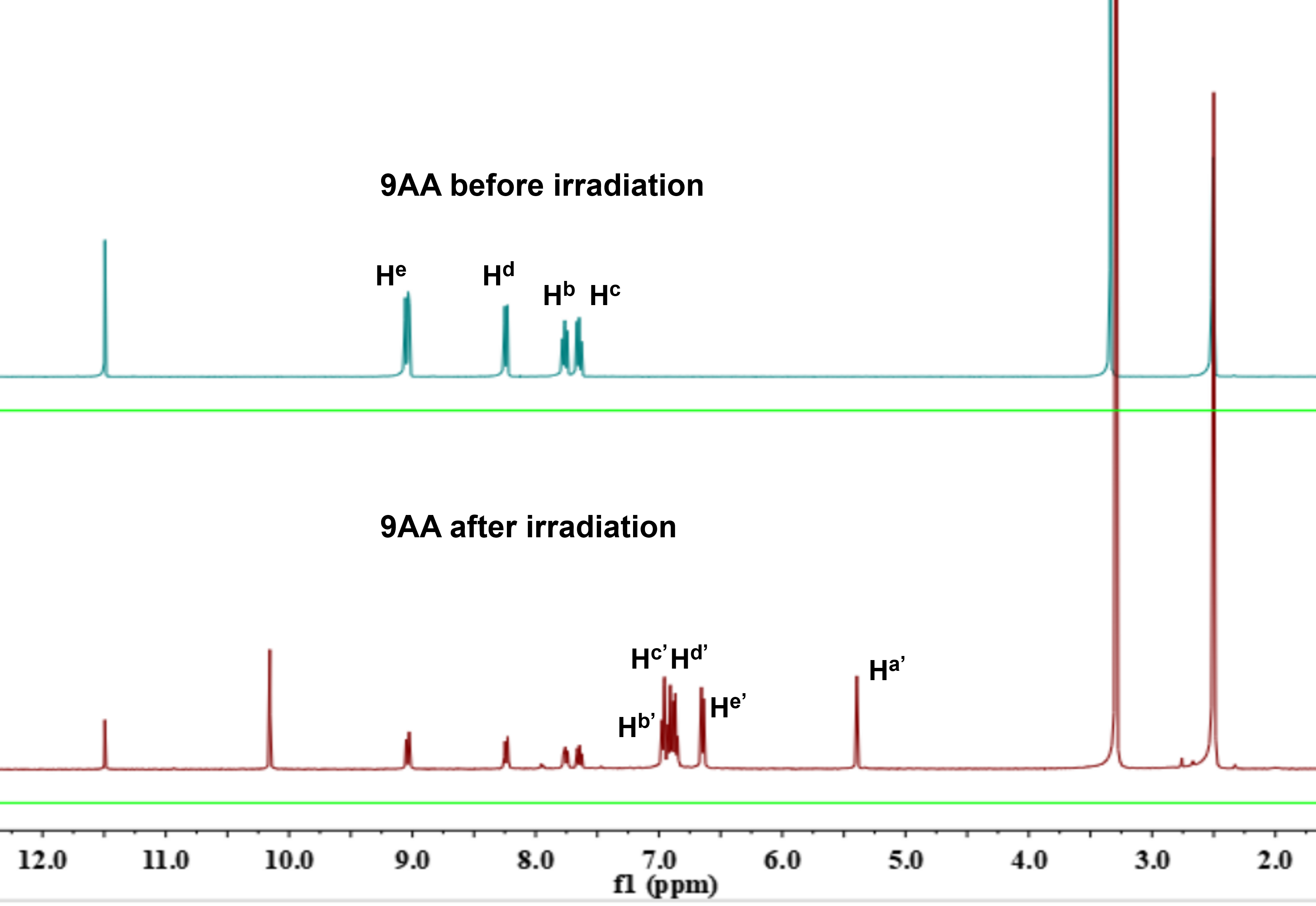


**Figure S20**. ^1^H NMR spectra of **9AA** before irradiation (top), after irradiation (bottom) with visible light for 3 days.


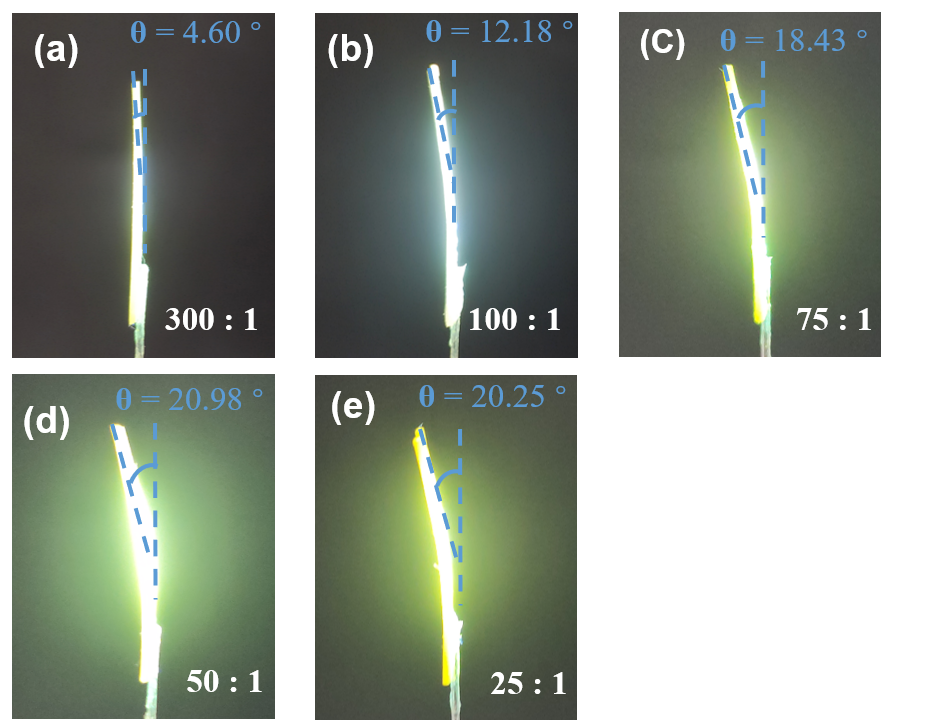


**Figure S21**. Comparison of photoresponsive bending behavior of **9EA@NSS-300** (a), **9EA@NSS-100** (b), **9EA@NSS-75** (c), **9EA@NSS-50** (d), and **9EA@NSS-25** (e) respectively. (membrane length, 2.0 cm; width, 5.0 mm, thickness, 450.0 µm.


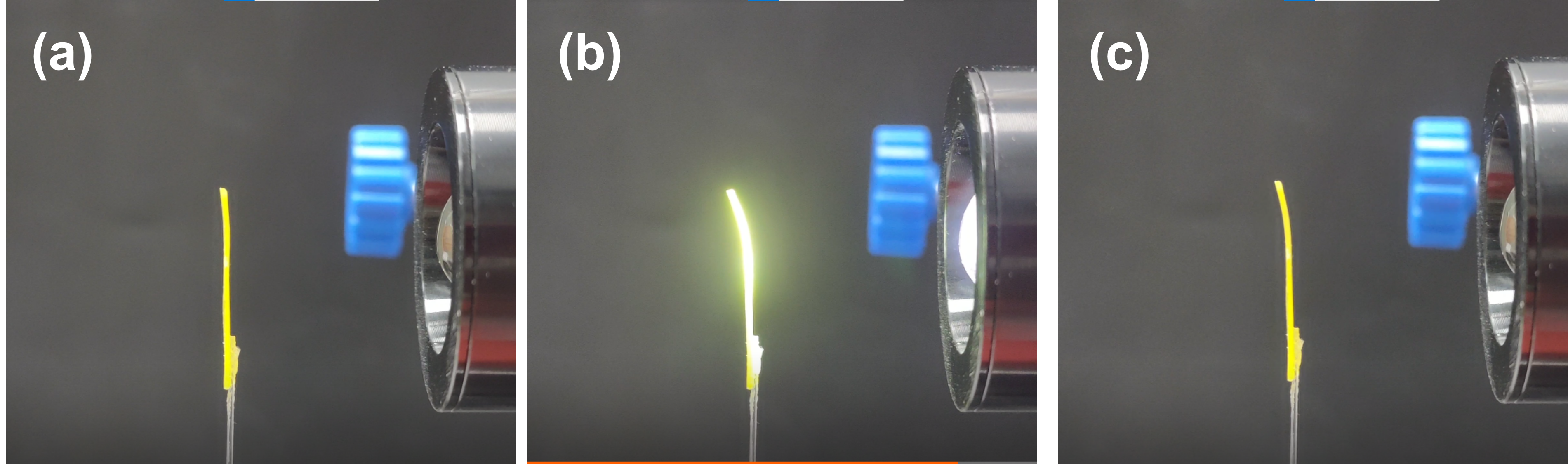


**Figure S22**. **9EA@NSS-50** actuator before (a) and after (c) 30 times photoresponse (b) within 5 minutes.


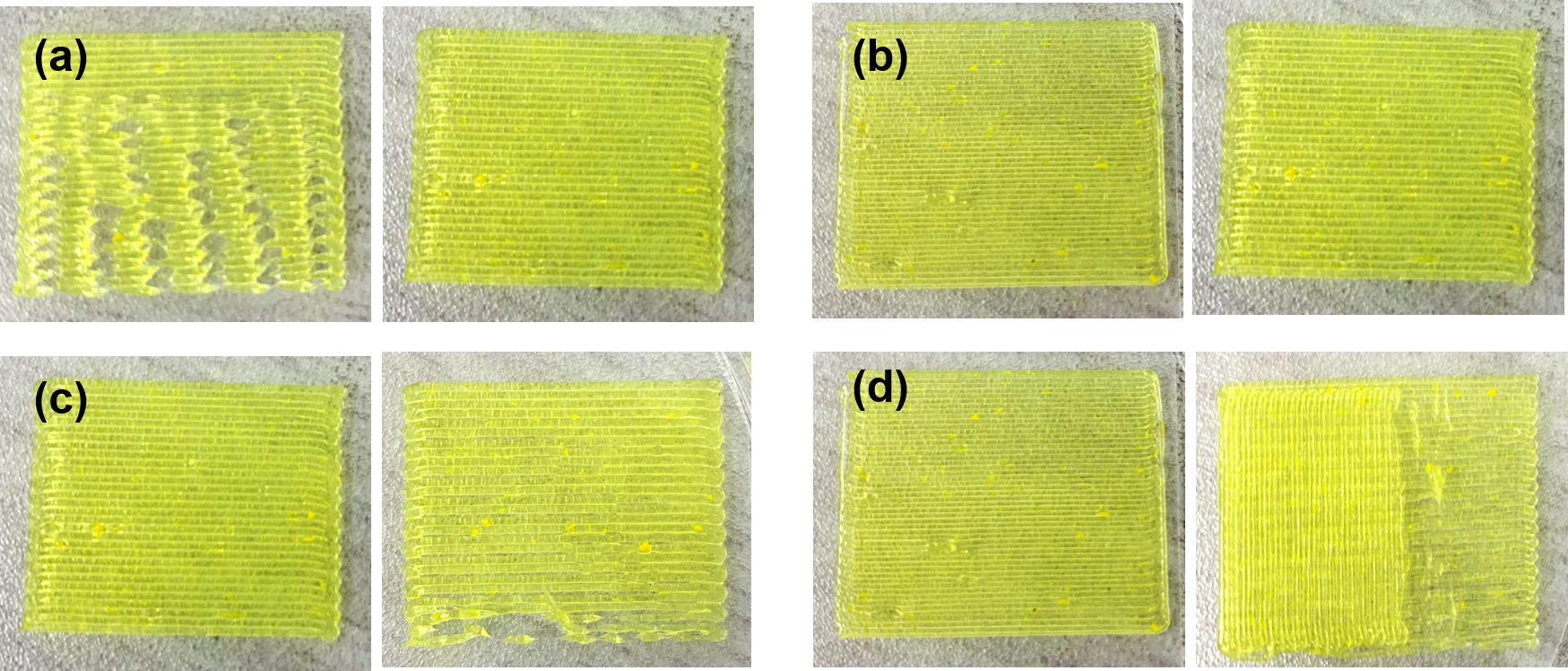


**Figure S23**. The digital image of the influence of different printing pressures (a), needle diameters (b), extrusion rates (c), and number of printing layers (d) on the printed material's morphology.


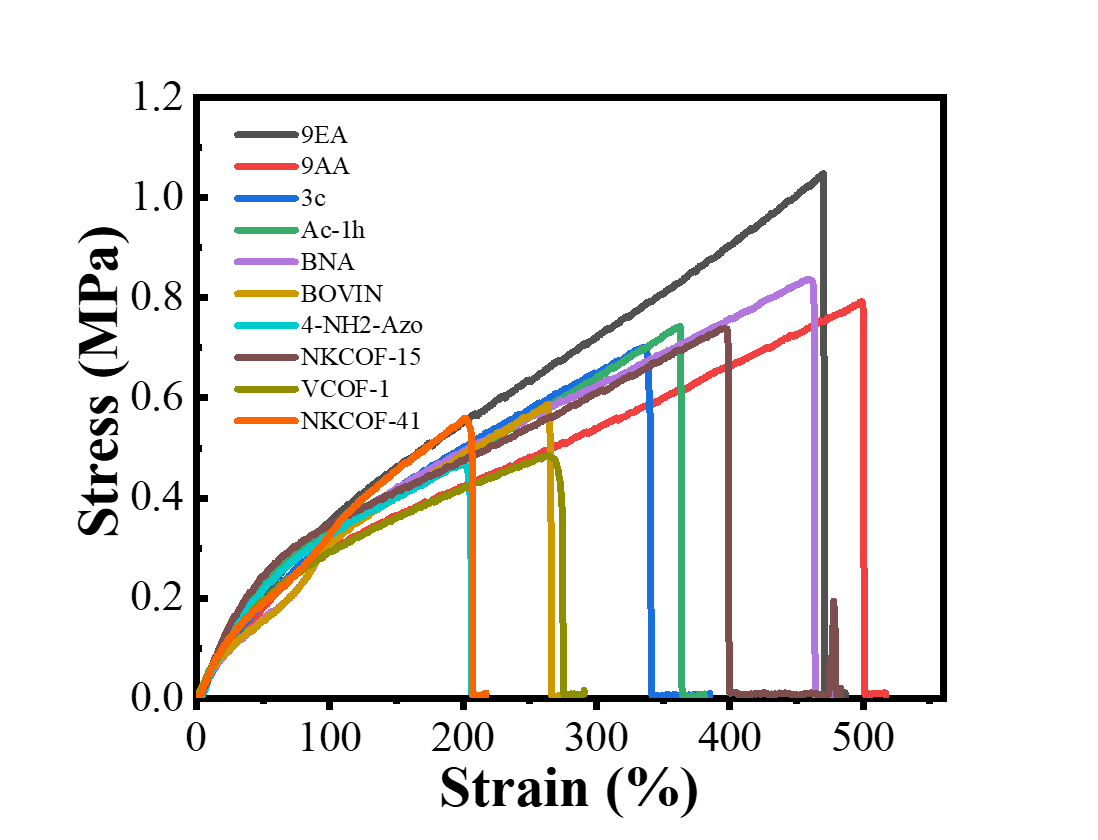


**Figure S24**. Stress−strain curves of different p-crystals @NSS-50 actuator (membrane length, 2.0 cm; width, 5.0 mm, thickness, 450.0 µm)


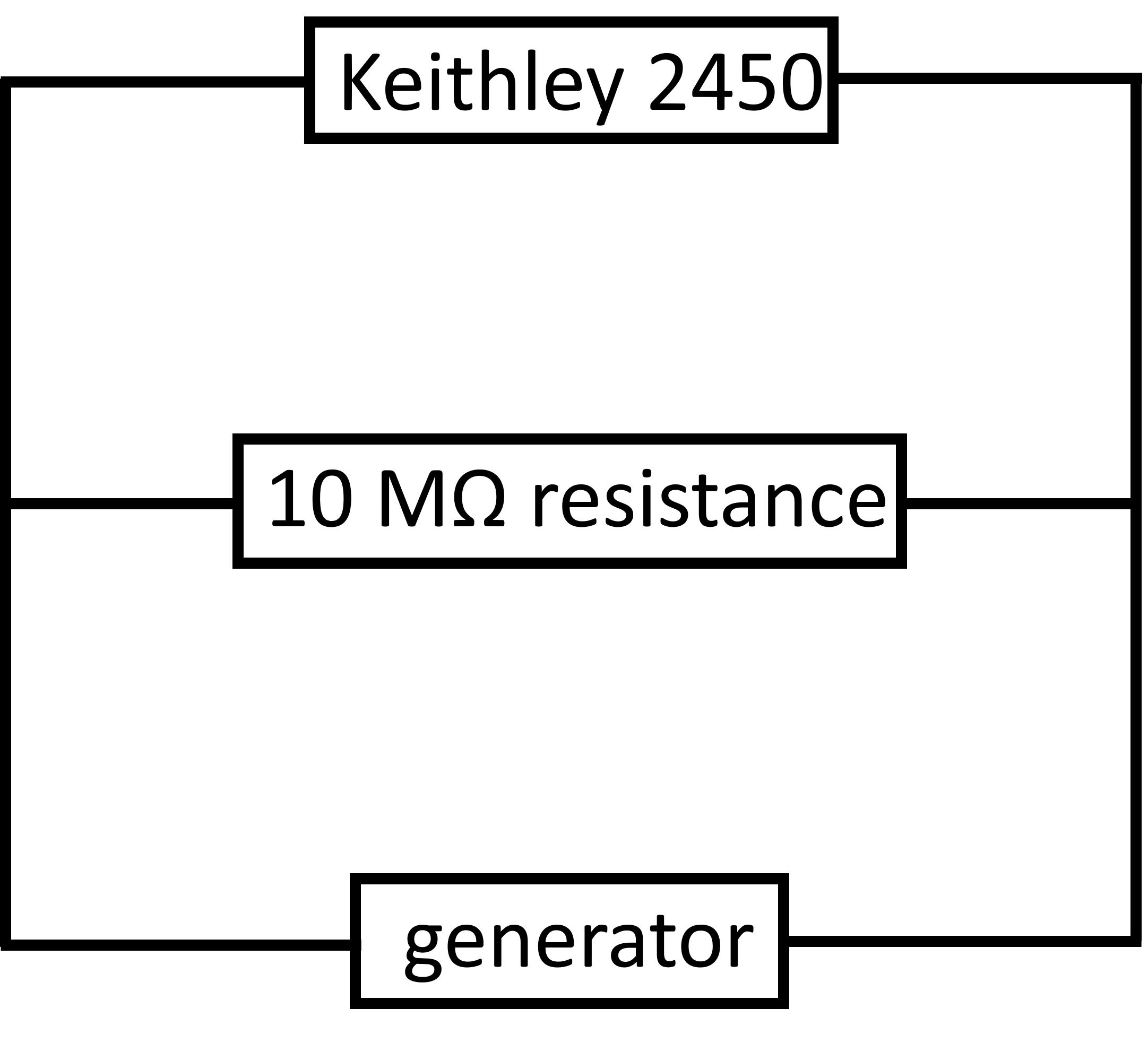


**Figure S25**. Schematic diagram of generator assembly.


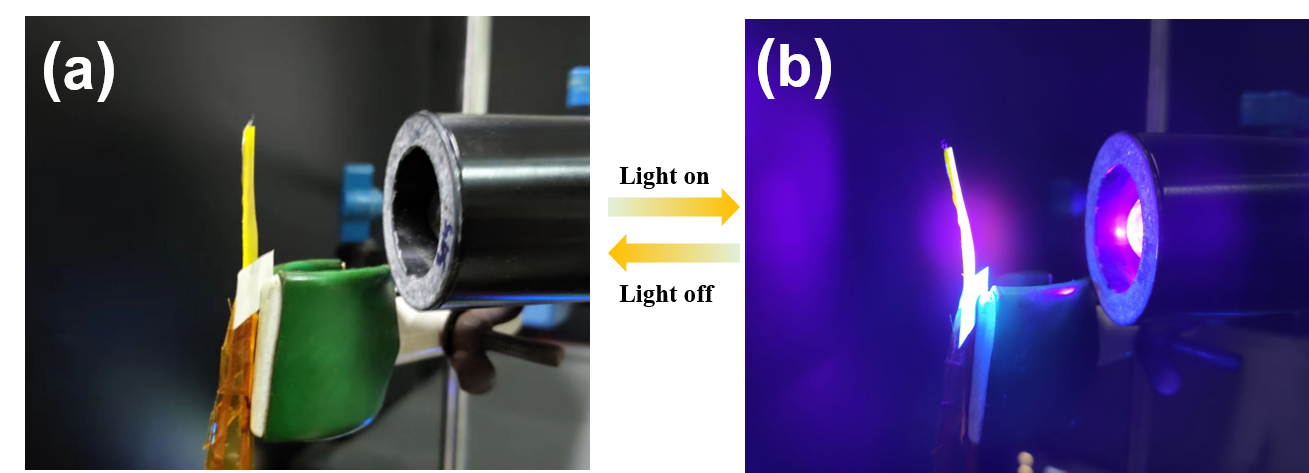


**Figure S26**. Photographs of the p-crystals@NSS@PVDF bilayer generator upon exposure to light stimulation.

**SI-3. Supplementary tables**

**Supplementary Table S1. Crystal data of Ac-a.**

|  | **Ac-a** |
| --- | --- |
| Formula | C_12_H_9_ClN_2_OS |
| CCDC number | 2356358 |
| Crystal system | monoclinic |
| Space group | *P 2_1_/c* |
| a/Å | 19.0825 (6) |
| b/Å | 12.9016(4) |
| c/Å | 9.6906(3) |
| α/° | 90 |
| β/° | 97 |
| γ/° | 90 |
| Volume/Å^3^ | 2366.(13) |
| Z | 8 |
| ρ_calc_ g/cm^3^ | 1.486 |
| μ/mm^‑1^ | 4.377 |
| Final R indexes | R_1_ = 0.0769, wR2 = 0.2287 |
| GoF | 1.112 |
